# Calcium carbonate precipitating extremophilic bacteria in an Alpine ice cave

**DOI:** 10.1038/s41598-024-53131-y

**Published:** 2024-02-01

**Authors:** Nóra Tünde Lange-Enyedi, Péter Németh, Andrea K. Borsodi, Christoph Spötl, Judit Makk

**Affiliations:** 1 Institute for Geological and Geochemical Research, HUN-REN Research Centre for Astronomy and Earth Sciences, Budaörsi út 45, Budapest, 1112 Hungary; 2 Department of Microbiology, Institute of Biology, Faculty of Science, ELTE Eötvös Loránd University, Pázmány P. sétány 1/C, Budapest, 1117 Hungary; 3 Research Institute of Biomolecular and Chemical Engineering, Nanolab, University of Pannonia, Egyetem út 10, Veszprém, 8200 Hungary; 4 Institute of Aquatic Ecology, HUN-REN Centre for Ecological Research, Karolina út 29, Budapest, 1113 Hungary; 5 Institute of Geology, University of Innsbruck, Innrain 52, 6020 Innsbruck, Austria

**Keywords:** Ecology, Microbiology, Biogeochemistry

## Abstract

Extensive research has provided a wealth of data on prokaryotes in caves and their role in biogeochemical cycles. Ice caves in carbonate rocks, however, remain enigmatic environments with limited knowledge of their microbial taxonomic composition. In this study, bacterial and archaeal communities of the Obstans Ice Cave (Carnic Alps, Southern Austria) were analyzed by next-generation amplicon sequencing and by cultivation of bacterial strains at 10 °C and studying their metabolism. The most abundant bacterial taxa were uncultured Burkholderiaceae and *Brevundimonas* spp. in the drip water, *Flavobacterium*, *Alkanindiges* and *Polaromonas* spp. in the ice, *Pseudonocardia*, *Blastocatella* spp., uncultured Pyrinomonadaceae and Sphingomonadaceae in carbonate precipitates, and uncultured Gemmatimonadaceae and Longimicrobiaceae in clastic cave sediments. These taxa are psychrotolerant/psychrophilic and chemoorganotrophic bacteria. On a medium with Mg^2+^/Ca^2+^ = 1 at 21 °C and 10 °C, 65% and 35% of the cultivated strains precipitated carbonates, respectively. The first ~ 200 µm-size crystals appeared 2 and 6 weeks after the start of the cultivation experiments at 21 °C and 10 °C, respectively. The crystal structure of these microbially *induced* carbonate precipitates and their Mg-content are strongly influenced by the Mg^2+^/Ca^2+^ ratio of the culture medium. These results suggest that the high diversity of prokaryotic communities detected in cryogenic subsurface environments actively contributes to carbonate precipitation, despite living at the physical limit of the presence of liquid water.

## Introduction

Bacteria thrive at temperatures under −10 °C in permafrost environments^1^ and above 100 °C in hydrothermal vents^2^. Psychrophiles, growing at temperatures < 20 °C with an optimum < 15 °C, and psychrotolerant microorganisms, growing at 0 °C with an optimum at 20–40 °C, belong to the group of extremophiles. Permanently frozen areas provide habitats for diverse taxonomical groups^3,4^. Understanding these cold-adapted microbes led to the discovery and biotechnological application of enzymes that are active at low temperatures, e.g., in the degradation of hydrocarbons^5,6^.

Permanently frozen solid material may contain pores and microchannels with liquid water and dissolved matter that might be utilized by bacteria. Similar conditions persist in Alpine (> 1500 m) caves that may contain perennial ice, e.g., due to their unique microclimate associated with the so-called chimney effect^7^. Ice caves represent extreme environments for bacteria due to the permanent cold temperature (< 1 °C), high Ca^2+^ (and locally also Mg^2+^) concentrations, low amounts of organic matter and desiccation stress on the cave walls. A few studies analyzed the bacterial communities of ice caves with next-generation amplicon sequencing methods, which provided insights into the abundant microbial lineages in these subterranean ecosystems. In particular, ice caves in the volcanos of Mauna Loa (Hawaii)^8^ and Mt. Erebus (Antarctica)^9^ as well as photic karst caves including Scărişoara Ice Cave (Romania)^10,11^, Paradana Ice Cave (Slovenia)^12^, Morgana ice Cave (Italy)^13^ and ice cave A294 (Spain)^14^ were investigated^15^. These caves are mostly inhabited by members of the phyla Pseudomonadota (previously Proteobacteria) and Actinomycetota (previously Actinobacteria) with lower proportions of Acidobacteriota (previously Acidobacteria), Bacteroidota (previously Bacteroidetes), Cyanobacteria, Bacillota (previously Firmicutes) and Gemmatimonadota (previously Gemmatimonadetes)^8–14^ based on molecular clone library^9^ and next-generation amplicon sequencing studies^8,10–15^. In ice caves diverse autotrophic and heterotrophic metabolic activities of carbon, nitrogen, sulfur, and iron cycles were reported based on metabarcoding and proteomic analysis of bacterial strains^8–14^. These activities are similar to moderate temperature karst caves, where microbiota participate in methane oxidation, heterotrophic ammonification and autotrophic nitrification^16–21^. However, mostly columnar ice and frozen lakes were examined in karst caves, and there is no information about the microbial communities inhabiting carbonate precipitates, cave walls or clastic sediments. Psychrophilic bacterial strains were cultivated from Eisriesenwelt Ice Cave^22^ and Hundsalm Eis- und Tropfsteinhöhle (both in Austria)^23^ as well as Scărişoara Ice Cave (Romania)^24^, but their calcium carbonate precipitating capability has not been examined.

In moderate temperature karst caves (~ 10 °C) and in polar environments (~ 0 °C) bacteria actively participate in the precipitation of calcium carbonate polymorphs, i.e., calcite, vaterite and aragonite^25–30^. Cell surface molecules including cell wall and extracellular polymer substance (EPS) *influence* calcium carbonate precipitation during carbonate crystal nucleation and metastable phase formation^27^. Psychrophilic bacteria adapt to low and near-freezing temperatures by modifying their cell wall and EPS compositions. In particular, the lipid (peptidoglycan) and choline contents of the cell wall as well as the polysaccharide and protein ratio of the EPS vary with incubation temperature^31^. The major metabolic processes involved in microbially *induced* carbonate precipitation are autotrophic photosynthesis, ammonification, ureolysis, dissimilatory nitrate and sulfate reduction as well as methane oxidation^17,28^. Experiments performed at 40 °C suggest that an increase of the Mg^2+^/Ca^2+^ ratio from 1 to 12 in the liquid experimental medium affects the Mg-content of the precipitates^32,33^. However, no information is available about solid experimental media, which would be necessary to understand carbonate precipitation on solid colonization surfaces similar to speleothems. Furthermore, most of the cultivation experiments were conducted at room or higher incubation temperatures, while few studies explored the carbonate precipitation process induced by the metabolic activity of bacteria at low (< 10 °C) temperatures^25,34–36^.

In order to explore the characteristics of psychrophilic bacterial and archaeal communities inhabiting cryogenic environments, we investigated samples from Obstans Ice Cave, situated in the Carnic Alps (Austria). The aim was to examine the microbial community in relation to the extreme conditions in this cryospheric environment. We (i) studied the abundance and morphology of biofilms on speleothems, (ii) explored the composition of bacterial and archaeal communities in various cave habitats including drip water, ice, carbonate precipitates and clastic cave sediment using next-generation amplicon sequencing methods, (iii) cultivated bacterial strains from speleothem surfaces using subsurface-environment imitating culture media at an incubation temperature of 10 °C and analyzed their metabolic characteristics in relation to oligotrophy, and (iv) tested if temperature and the Mg^2+^/Ca^2+^ ratio influence calcium carbonate precipitation and carbonate polymorph selection.

## Results and discussion

### Description of the sampling site in the Obstans Ice Cave

Samples were collected in the Obstans Ice Cave, known from its modern aragonite formation at near-freezing conditions^37^. The cave opens at 2174 m a.s.l. in the north-facing cliff (46.6875° N and 12.4932° E) and comprises a network of 3364 m of galleries with a vertical extent of 139 m. The mean annual air temperature in the cave is 1.0 ± 0.4 °C and perennial ice is present a few tens of meters behind the entrance^38^. The cave developed in Devonian limestones which contain thin dolomitic layers. Cave drip water is characterized by a pH of 8.5, an electric conductivity of 414–577 μS/cm, and 41–58 mg/l Mg^2+^, 32–48 mg/l Ca^2+^, 222–275 mg/l HCO_3_^-^, and 69–232 mg/l SO_4_^2−^. Modern aragonite precipitates as flowstone, stalagmites and stalactites along with low Mg-calcite and hydromagnesite^37^. It is of interest that an aragonite precursor phase, monoclinic aragonite (mAra), has been reported from this cave^39^. Older, inactive speleothems consist of low-Mg calcite and/or aragonite^38^.

**Figure 1:**
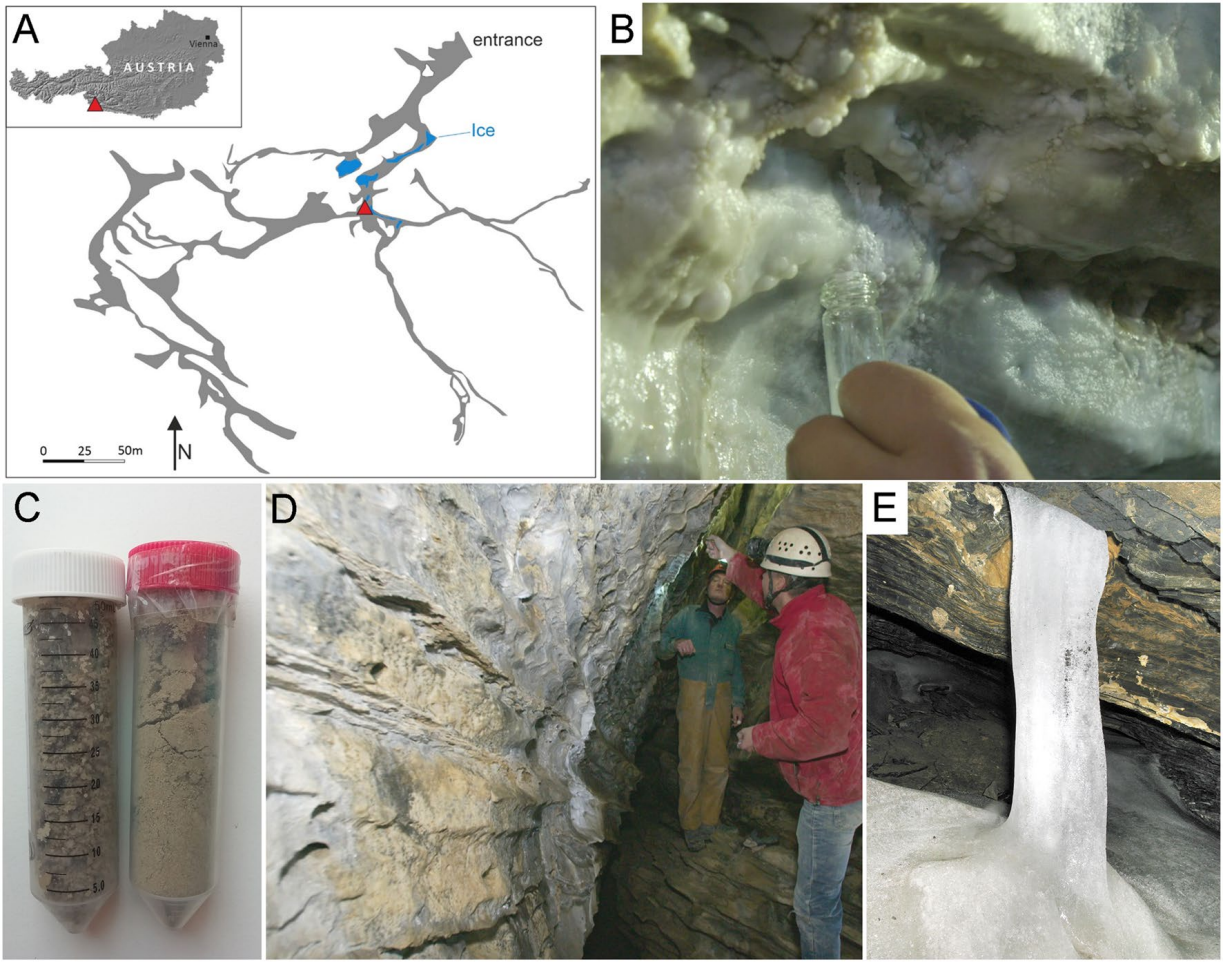
Samples and sampling sites in the Obstans Ice Cave: (**A**) Location and plan view of the cave (based on Spötl et al.^37^) with the sampling site (red triangle), (**B**) aragonite coating the cave wall (sample OAR), (**C**) fine-grained clastic cave sediment in 3 cm diameter plastic containers (samples OSE1-2), (**D**) surface of cave wall without carbonate coating (sample OCW), and (**E**) ice column (sample OIC). The width of the column is 30 cm. Informed consent was obtained to publish this figure in an online open access publication.

Previous studies have shown that microbial communities of caves are distributed along gradients of different variables including distance from the entrance, depth, habitats of the drip water and the mineralogical composition of speleothems, cave walls and clastic sediments^17,18,40^. Their diversity may also vary between chambers, and multiple samples are necessary to document their distribution and role in the local biogeochemical processes^17,18,40^. Here, we focused on microbial samples from one gallery, approximately 50 m behind the entrance of the cave, where mAra precipitation was previously documented (Fig. 1). Seven samples were taken to investigate the microbial community of aragonite (OAR) compared to control samples including cave walls with calcite coating (OCA) and without carbonate (OCW), clastic sediments (OSE1 and OSE2), drip water (ODW) and ice (OIC). The OSE1 and OSE2 samples contained quartz, clay minerals, small amounts of plagioclase, Mg-calcite, chlorite, and pyrite.

### Bacterial and archaeal taxonomic composition of the Obstans Ice Cave

**Figure 2:**
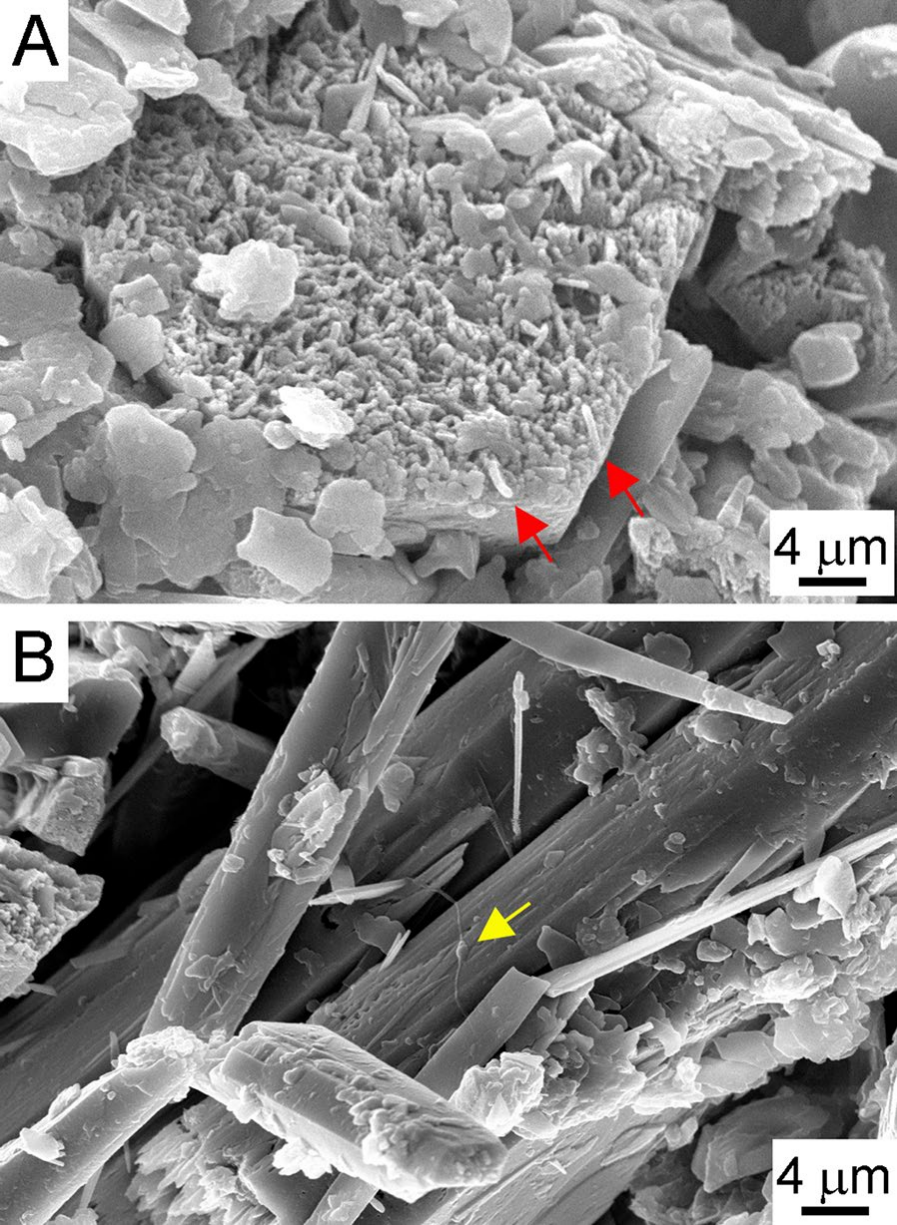
SEM micrographs of 5% glutaraldehyde-fixed and lyophilized surface samples from Obstans Ice Cave: (**A**) hydromagnesite and (**B**) aragonite crystals with rods (red arrows) and stalked prokaryotic cells (yellow arrow).

Scanning electron microscopy (SEM) images reveal only scarce elongated prokaryotic cells on the surface of hydromagnesite (Fig. 2A) and needle-shaped aragonite (Fig. 2B). Altogether 217,063 bacterial and 15,230 archaeal sequences were detected by amplicon sequencing (Table 1, Supplementary Table S1–3 online), which correspond to 3666 bacterial and 55 archaeal OTUs, respectively. The Good's values were close to 100% suggesting high sequence coverage. The highest diversity values were found in the ODW and OCW samples, while OIC and OSE2 proved to host the least diverse communities (Table 1).

**Table 1:** Sequence numbers, OTU-numbers, coverage, estimated species numbers and diversity indices of bacterial communities in the Obstans Ice Cave.

Sample	Number of reads	Number of OTUs	Good's coverage (%)	Chao1	Shannon	Inverse Simpson's (1/D)
ODW	40,411	1683	98	1722 (1674; 1785)	4.87 (4.83; 4.91)	19.6 (18.9; 20.3)
OIC	18,263	530	99	530 (530; 530)	3.31 (3.28; 3.34)	11.1 (10.8; 11.3)
OAR	43,262	511	99	535 (502; 587)	4.29 (4.27; 4.31)	34.4 (33.4; 35.3)
OCA	35,052	802	99	864 (819; 927)	4.47 (4.45; 4.50)	39.5 (38.4; 40.6)
OCW	27,536	753	99	773 (755; 803)	4.67 (4.64; 4.70)	41.4 (40.2; 42.7)
OSE1	22,112	534	99	538 (534; 549)	4.43 (4.40; 4.45)	31.4 (30.4; 32.4)
OSE2	30,427	237	99	246 (234; 272)	3.23 (3.21; 3.25)	13.1 (12.8; 13.3)

The estimated species numbers and diversity indices were based on 18,263 subsampled sequences. Values corresponding to lower and upper limits of 95% confidence intervals are given in parentheses. Sample abbreviations are *OAR* Obstans aragonite, *OCA* Obstans calcite, *OCW* Obstans cave wall, *ODW* Obstans drip water, *OIC* Obstans ice, and *OSE1–2* Obstans sediment.

**Figure 3:**
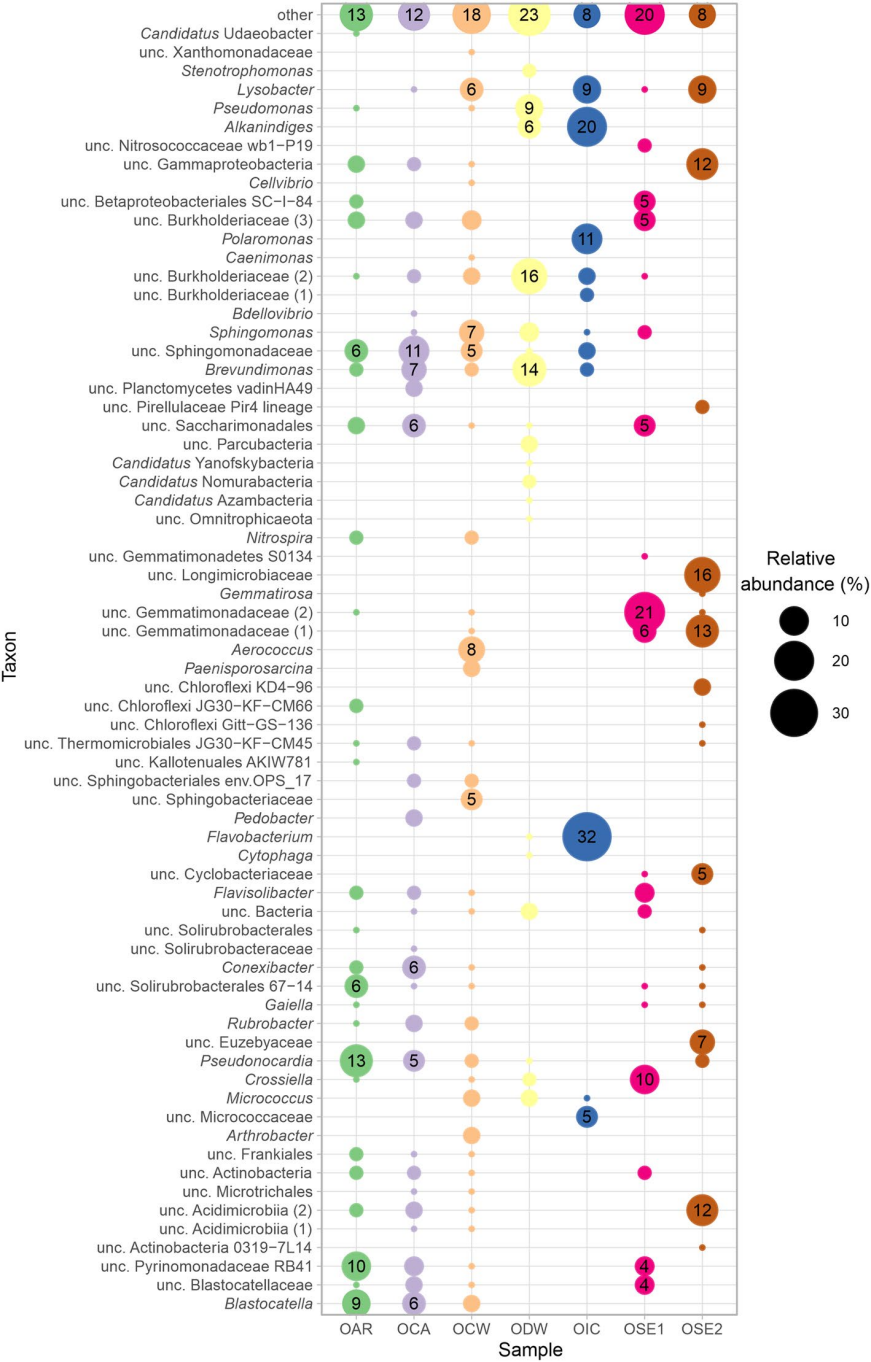
Distribution of bacterial OTUs among genera, based on the amplicon sequencing of V3-V4 regions of 16S rRNA gene sequences, of the Obstans Ice Cave above 1% relative abundance. Sample abbreviations are *OAR* Obstans aragonite, *OCA* Obstans calcite, *OCW* Obstans cave wall, *ODW* Obstans drip water, *OIC* Obstans ice, and *OSE1–2* Obstans clastic sediment, *unc* uncultured.

The bioinformatical analyses showed similar sets of dominant taxa in different habitats, although large differences were found in their abundance (Fig. 3, Supplementary Table S1 online). The main habitat types, i.e., cave wall surfaces (OAR, OCA, OCW), water (ODW, OIC) and clastic sediments (OSE1–2) differed. Most sequences obtained are either previously unknown bacterial genera or of higher taxonomic level. Most of these uncultured bacterial sequences were detected in the OSE1–2 samples, and most of the well-described genera were found in the ODW and OIC samples.

Despite the extreme conditions prevailing in the Obstans Ice Cave, a diverse microbial community was observed. These bacteria adapted to the cold temperature, high Ca^2+^ and Mg^2+^ concentrations, presumably low amounts of nutrients and desiccation stress. Pseudomonadota were present in large proportions (22–58%), especially in the ODW and OIC samples. The most abundant genera were *Alkanindiges*, uncultured Burkholderiaceae and *Brevundimonas*. Bacteria commonly described as oligocarbophilic organisms, such as *Sphingomonas*^41^ and *Brevundimonas*^42^, were also identified. The described cultivated members of *Polaromonas* usually grow at temperatures below 15 °C^43^.

Actinomycetota were abundant in the surface biofilm (OAR, OCA, OCW: 23–35%) and in the clastic sediment (OSE1–2: 17–29%) samples. *Pseudonocardia* (OAR: 13%), uncultured Acidimicrobiia (OSE2: 12%) and *Crossiella* (OSE1: 10%) reached the highest abundance. The branched substrate mycelium-forming *Crossiella* was found in moonmilk deposits in caves around the word, and this genus could be expected to play an active role in the formation of moonmilk^16^. Spore formation characteristic of the family Pseudonocardiaceae might help survival under extreme environmental conditions such as low nutrient input or desiccation. Sequences affiliated with Acidobacteriota also reached relative abundance up to 6–20% in the OAR, OCA and OCW samples. Members of *Blastocatella* and the RB41 lineage of the family Pyrinomonadaceae were prominent in the OAR sample, though their potential metabolic role is unknown.

Members of Bacteroidota, especially *Flavobacterium,* dominated the OIC (32%) sample. This genus usually contains psychrophilic bacteria^44^. *Candidatus* Patescibacteria, commonly identified in karst cave samples by next-generation sequencing^16–21^, was mainly detected in the ODW sample (14% abundance). Gemmatimonadota were prominent in the OSE1–2 samples (30–31%) (Fig. 3). These bacteria were often detected in hyperarid deserts and are known to tolerate long-term desiccation^45^. Bacillota was mostly represented by the facultative anaerobic *Aerococcus* in the OCW (8%) sample. In contrary to the high number of previously unknown bacterial genera or higher taxonomic level, several sequences are closely related (> 97% sequence similarity) to bacteria inhabiting cold environments. For example, OTU25 (uncultured Solirubrobacterales 67–14) was also detected in Antarctic soils^46^.

The less abundant and diverse archaeal communities of surface biofilms contained mostly Euryarchaeota (uncultured Thermoplasmata, 98–100%). The archaeal OTU2 (Thermoplasmata) is part of the microbiota inhabiting permafrost soils in acidic wetlands of the Canadian High Arctic^47^. The OSE1 and OIC samples contained members of Nanoarchaeaeota at 91% and 54% abundance, respectively. These bacteria with small genome and cell size can adapt to extreme environmental conditions, similar to *Candidatus* Patescibacteria^48^. Sequences belonging to Nitrososphaerota (previously Thaumarchaeota) were abundant in the OIC sample (16%) (Supplementary Fig. S1, Table S2 online).

### Cultivable bacterial diversity of the Obstans Ice Cave

Samples from cave wall surfaces (OCW, OAR and OCA) and clastic cave sediment (OSE1) were chosen for cultivation at 10 °C to study microbially *induced* calcium carbonate precipitation. The R2A, 10% R2A, CM and B4 media were previously successfully applied to cultivate diverse bacterial strains, adapted to the high Mg^2+^ and Ca^2+^ concentrations of the karst environments^28^, therefore we applied them also in this study. The OB4M1.5 medium had a lower glucose content than B4, but it was adjusted with MgSO_4_ to reach the same Mg^2+^/Ca^2+^ molar ratio (1.5) as the drip water of the Obstans Ice Cave. We also decreased the organic matter content with the application of 10% R2A.

Altogether 110 bacterial strains were isolated from the ice cave samples, and they showed > 97% 16S rRNA gene sequence similarity to the closest bacterial species (Supplementary Table S4 online). The bacterial strains were affiliated with 44 species, 21 genera in the phyla Bacillota (47%), Actinomycetota (35%), Pseudomonadota (15%) and Bacteroidota (3%). The most frequently isolated strains were members of genera *Bacillus, Arthrobacter* and *Paenisporosarcina*. The members of genera *Pseudomonas, Flavobacterium* and *Brevundimonas* were successfully cultivated, and they represent 9–16% relative abundance of the amplicon sequences of the ODW and OIC samples (Fig. 3). The genera *Arthrobacter*, *Micrococcus* and *Paenisporosarcina* also occurred in small proportions (1–5%) in the communities. The applied media were thus appropriate to culture several heterotrophic bacteria, which could be active members of the Obstans Ice Cave’s microbiota. The morphology of *Brevundimonas* spp*.,* observed on the surface of aragonite (Fig. 2), was similar to the prosthecate cells possessing appendages. Most of the genera were detected on the low organic matter-containing CM medium. Only two genera could be cultivated on the highest organic matter-containing B4 and R2A media (Supplementary Table S4 online). The excess organic matter allowed the growth of the more competitive genera of *Priestia* and *Peribacillus* (previously *Bacillus*) instead of oligotrophic bacteria. The highest Mg^2+^-containing OB4M1.5 medium enhanced the growth of Mg^2+^-tolerant or -dependent bacterial strains (Supplementary Table S4 online). For example, the high abundance of the OCW-111 strain was observed on the OB4M1.5 medium, similar to its close relative *Planococcus salinarum*, which also requires Mg^2+^ salts to grow^49^. Most of the identified genera were isolated from the OCA sample, which was also the most diverse of the solid samples based on amplicon sequencing (Table 1).

Close relatives of extremophilic bacteria were found among the cultivated bacterial strains. Examples include psychrophilic and psychrotolerant bacteria such as *Devosia psychrophila*^50^ (Supplementary Table S4 online). The members of halotolerant bacteria, i.e., strains that tolerate high inorganic salt concentrations, were also cultivated. The close relative of the OCW-103 strain, *Pseudomonas extremaustralis* 14-3, was isolated from an ephemeral pond in Antarctica and showed resistance to heat, oxidative and cold stress, and accumulated polyhydroxy-butyrate as carbon storage in oligotrophic environments^51^.

In most karst caves, numerous biogeochemical processes operate, including steps in the carbon and the nitrogen cycle^18,19,40^. In our study, oxidation of glucose was detected in 33% of the strains, but fermentation of glucose was only found in one strain (*Hymenobacter tenuis* OCW-101). Biochemical tests (Supplementary Table S5 online) demonstrated that 50% of the tested strains hydrolyze urea, 75% produce nitrite, 50% ammonia and 58% were positive for nitrogen production from nitrate, i.e., they are active in the nitrogen cycle. The ammonification test using peptone media showed that 58% of the strains were positive. These metabolic activities are known to be also important processes for microbially *induced* calcium carbonate precipitation^25–28^.

### Comparison of the microbial communities of the Obstans Ice Cave with other ice caves

Two studies examined microbial communities of ice caves formed in basaltic bedrock (Supplementary Table S6 online). Ice and calcite, gypsum and silica precipitates in caves of the Mauna Loa volcano (Hawaii) contained only a few genera (*Pedobacter*) in common with the Obstans Ice Cave^8^. Molecular clones of the Mt. Erebus volcanic ice caves (Antarctica) were reported to be dominated by Acidobacteriota in the Fe- and Mn-containing cave sediments with the members of *Blastocatella* and the unc. Pyrinomonadaceae RB41. *Sphingomonas* and the unknown Solirubrobacterales and Burkholderiales lineages were also common in the Obstans Ice Cave. Phototrophic Cyanobacteria and Chloroflexota were abundant in ice blocks exposed to sunlight in the Mt. Erebus caves^9^. Cyanobacteria and Chloroflexota also showed high abundance in illuminated areas of ice blocks in Scărişoara Ice Cave (Romania) according to pyrosequencing^10^ and Illumina^11^ next-generation amplicon sequencing data (Supplementary Table S6 online). In contrast, the sampled gallery in the Obstans Ice Cave is a completely aphotic environment, therefore the detected Chloroflexi lineages could be facultative or obligate heterotrophic bacteria. The varying abundance of Pseudomonadota and Actinomycetota members at different times of the year in Scărişoara Ice Cave indicates that allochthonous organic matter content is important for the development of microbial communities in ice caves. The common genera in the Obstans Ice Cave, especially in the OIC and the ODW samples, contained many heterotrophic bacteria including *Pseudomonas*, *Stenotrophomonas, Brevundimonas, Pseudonocardia, Polaromonas, Flavobacterium, Pedobacter*^10,11^ (Supplementary Table S6 online). Furthermore, the close relatives of several cultivated bacteria including *Pseudomonas, Arthrobacter, Flavobacterium, Bacillus, Paenibacillus* and *Sporosarcina* were also reported from Scărişoara Ice Cave^24^. These genera involve aerobic chemoorgano-heterotrophic bacteria that can be easily cultivated (Supplementary Table S1 online, Fig. 3). Some bacteria in the Obstans Ice Cave such as *Alkanindiges*^52^ can also decompose complex polymers such as plant (lignin, cellulose) and animal (chitin) matter originating from soils.

The microbiota of a frozen lake in the Paradana Ice Cave (Slovenia) was the most similar to the Obstans Ice Cave. The previously mentioned heterotrophic genera, *Gaiella* and *Lysobacter* were also detected in Paradana Ice Cave^12^. A few common genera, *Lysobacter, Sphingomonas Pedobacter, Arthrobacter* were observed in the microbiota of the A294 Ice Cave (Central Pyrenees, Iberia), which mainly consisted of Pseudomonadota, Bacteroidota and Actinomycetota^14^. Therefore, the microbiota of ice and drip water samples from ice caves formed in limestone bedrock show similarities and presumably contain more heterotrophic bacteria from the surface than the habitats found on the surface of precipitates and clastic sediments. The vermicular deposits in Morgana Cave (Italy) also contain abundant Pseudomonadota, such as unc. wb1-P19, unc. Nitrosomonadaceae and unc. Burkholderiaceae. Uncultured Acidimicrobiia, unc. Rubrobacteria, *Gaiella* (Actinomycetota), unc. Blastocatellaceae and unc. Pyrinomonadaceae (Acidobacteriota) were abundant in vermicular deposits containing dolomite and clay minerals^13^ (Supplementary Table S6 online). The genera *Crossiella, Rubrobacter, Conexibacter, Paenisporosarcina, Aerococcus, Gemmatirosa, Nitrospira, Bdellovibrio, Caenimonas, Alkanindiges* and *Candidatus* Udaeobacter (> 1% relative abundance) identified in samples from Obstans Ice Cave are, however, new members of the microbial community of ice caves (Fig. 3).

Low number of archaeal sequences were detected in nearly every previously examined cave^8,9,11,13^ (Supplementary Table S6 online), consistent with the previous study of moonmilk deposits from the Obstans Ice Cave^53^. The phyla Euryarchaeota^8,11^ and Nanoarchaeota^13^ were the dominant members of archaeal sequences in ice caves formed in limestone bedrock. Nitrososphaerota are notably more abundant in warmer (> 10 °C) caves^18,19,40^ than in the cold Obstans Ice Cave. Presumably methanogenic bacteria were detected in the classes Methanomicrobia and unc. Thermoplasmata (Euryarchaeota)^11,13^. Methanotrophic and methanogenic activities were also identified in Morgana Cave (Italy) with the presence of certain taxa that were not part of the microbiota from Obstans Ice Cave^13^. Multiple CO_2_-fixing, methanotrophic and H_2_-fixing pathways were detected by qPCR, indicating that autotrophic chemolithotrophy may be an important metabolic activity in basaltic caves in relation to volcanic emissions^9^. Ruiz-Blas et al.^14^ found multiple steps of the carbon, nitrogen, sulfur, and iron cycles based on metabarcoding and proteomic analysis of samples from the A294 Ice Cave (Spain). Beside the mentioned heterotrophs, the Obstans Ice Cave is also inhabited by known chemolithoautotrophes such as members of sediment iron (pyrite)-oxidizing uncultured Acidimicrobiia^13^, facultative ammonia-oxidizing *Pseudonocardia*^54^, ammonia-oxidizing *Candidatus* Nitrosocosmicus (Archaea)^17^, *Nitrosospira, Nitrosomonas* (Bacteria) and nitrite-oxidizing *Nitrospira*^17,18,20,21,55^. There may be differences in the metabolism of closely related species and genera, but the results of the biochemical tests (Supplementary Table S5 online) suggest several steps in the nitrogen cycle in Obstans Ice Cave.

### Microbially induced calcium and magnesium carbonate precipitating experiments

Several bacterial genera known to precipitate calcium carbonate were cultivated from the samples, including *Pseudomonas*, *Lysobacter, Brevundimonas, Micrococcus, Arthrobacter*^28^*, Janibacter*^56^, *Paenisporosarcina*^57^, *Planococcus*^58^, *Flavobacterium*^59^ and *Massilia*^26^. To test their calcium and magnesium carbonate precipitating activity, 23 bacterial strains were selected and incubated on solid carbonate precipitation media containing of only Ca^2+^ (B4) or equal concentrations of Mg^2+^ and Ca^2+^ (B4M1) for one year at 10 °C and 21 °C (Supplementary Table S7 online). The B4 medium has been used for testing the calcium carbonate precipitation activity of strains and for investigating the microbially *induced* carbonate precipitation mechanism^26,28,32,35^. Enyedi et al.^26,28,32,35^ reported that after inoculation of bacterial cells into Ca^2+^-containing medium, Ca^2+^ ions are bound by negatively charged and polarized functional groups present on bacterial surfaces. As they hydrolyse the yeast extract-originated amino acids, they produce ammonia, increase the pH of their microenvironment, and induce CaCO_3_ precipitation in the EPS in the form of amorphous calcium carbonate (ACC) within the first few days. As the bacterial cells disintegrate, enzyme release results in the local degradation of the ACC covering hydrophobic layers, which triggers the conversion of ACC to calcite^27^. Bacteria decrease the water activity of their environment by absorbing water during metabolism, while cultivated heterotrophic bacteria produce water and carbon dioxide through respiration, which can be incorporated into the carbonate precipitates.

All strains precipitated carbonate around the colonies in the medium on every type of agar plate. In the absence of Mg^2+^ (B4 medium) only 50% of the tested strains precipitated at 10 °C and 44% at 21 °C. On the B4M1 medium with Mg^2+^/Ca^2+^ = 1, 35% of the tested strains precipitated at 10 °C and 65% at 21 °C. Neither the elevated Mg^2+^content (Mg^2+^/Ca^2+^ = 1) nor the low incubation temperature (10 °C) stimulated carbonate precipitation in the bacterial biomass (Supplementary Table S7 online), in contrast to the results of Al Disi et al.^33^. Strains belonging to species *Arthrobacter glacialis, Hymenobacter tenuis, Jeotgalibacillus marinus, Pseudomonas extremaustralis, Paeniglutamicibacter sulfureus, Rhodococcus cerasti* and *Rhodococcus fascians* were identified as new calcium carbonate precipitating bacteria in our experiments*.*

Crystals larger than 200 µm appeared two weeks after the start of the experiment for most of the strains at 21 °C. In contrast, crystals of this size occurred only after 6 weeks when incubating the strains at 10 °C. According to tests with the cresol-red indicator, the highest alkalinization was reached after 9–20 days of incubation at 21 °C and after 26–83 days at 10 °C (Supplementary Tables S8–S9 online). These results show that the metabolic rate was higher at 21 °C than at 10 °C, consistent with previous studies^29,35^. Until the end of the incubation period, the amount of the precipitates was generally higher at 21 °C than at 10 °C (Supplementary Table S7 online). For a given strain, medium composition, temperature, the amount of CO_2_ and H_2_O released by respiration can be assumed to be the same. Rising temperatures increase respiration, i.e., the CO_2_-content, and the elevated carbonate concentration leads to supersaturation and carbonate precipitation.

**Figure 4:**
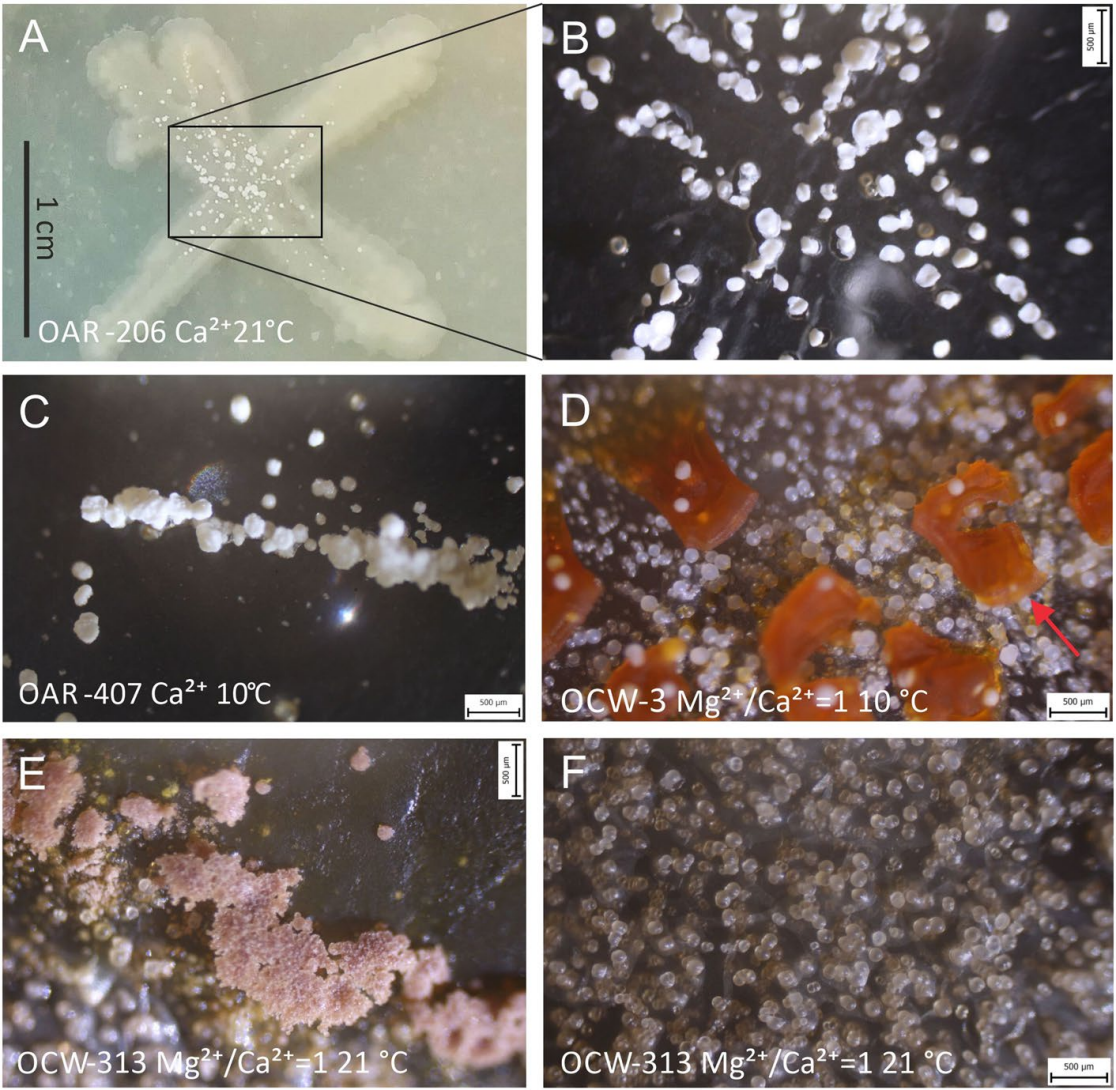
Stereomicroscopic images showing crystals after incubation for 26–52 weeks: (**A**) colony and (**B**) precipitates of *Peribacillus simplex* OAR-206 incubated at 21 °C on B4 medium, (**C**) *Peribacillus simplex* OAR-407 incubated at 10 °C on B4 medium, (**D**) *Rhodococcus fascians* OCW-3 incubated at 10 °C on B4M1 medium*,* (**E,F**) *Arthrobacter glacialis* OCW-313 incubated at 21 °C on B4M1 medium. Red arrow: remnant of the desiccated bacterial colony.

Spherulitic crystals (100–200 µm) were formed in every incubation condition (Fig. 4). Interestingly, these crystals formed aggregates on the edge of the colonies of *Arthrobacter glacialis* OCW-313 (Fig. 4E). Crystals precipitated after 26 weeks were identified by X-ray diffraction as calcite for most of the strains in the Mg^2+^-free experiments (Supplementary Table S7 online). However, the strain OCA-15 identified as *Paeniglutamicibacter sulfureus* precipitated vaterite in addition to calcite, similar to a *Paeniglutamicibacter* strain cultivated from Baradla Cave (Hungary) at 10 °C^28^. Mg-calcite precipitated from most of the strains in the Mg^2+^/Ca^2+^ = 1 experiments, and aragonite precipitation was observed only for the spherulitic crystals associated with the strain OCW-2 identified as *Arthrobacter glacialis* incubated at 21 °C and the strain OCW-101 identified as *Hymenobacter tenuis* incubated at 10 °C and 21 °C (Supplementary Fig. S2, Table S7 online). At Mg^2+^/Ca^2+^  > 2 the surface energy of aragonite decreases compared to Mg-calcite and thus aragonite precipitation is favored^60^.

EPS might have an important role on polymorph selection as previous experiments indicated an effect of the bacterial EPS on calcite, Mg-calcite and dolomite precipitation by crossing this thermodynamic barrier^33,61^. Al Disi et al.^33,61^ observed Mg-calcite and no aragonite, although they cultivated *Virgibacillus* strains at 20–40 °C on the solid MD medium characterized by particularly high Mg^2+^/Ca^2+^ ratios (6 and 12). Only Mg-calcite was detected for several bacterial species incubated at Mg^2+^/Ca^2+^ ~ 2 using the M2 medium^62^. Zhang et al. identified aragonite incubating the *Arthrobacter* sp. strain MF-2 in a liquid medium with Mg^2+^/Ca^2+^ = 6^32^. These results suggest that a high Mg^2+^ content is not a prerequisite for microbially *induced* aragonite precipitation, but it is a genus-dependent process.

We investigated the incorporation of Mg^2+^ into the precipitates of strain OAR-202, identified as *Peribacillus simplex,* by adding Mg^2+^ to the Ca^2+^-containing B4 medium at pH 7.5 and prepared media (B4; B4M0.25; B4M0.5; B4M1; B4M1.5) with Mg^2+^/Ca^2+^ ratios of 0, 0.25, 0.5, 1, 1.5. (see section “Carbonate precipitation experiments” in “Methods”). The latter molar ratio is similar to the drip water in Obstans Ice Cave. The Petri dishes were incubated at 21 °C, because the strains grow faster at this temperature than at 10 °C. Our data indicated that the mineralogical composition of the precipitates was similar at 10 °C and 21 °C (Supplementary Table S7, Fig. S2 online).

**Figure 5:**
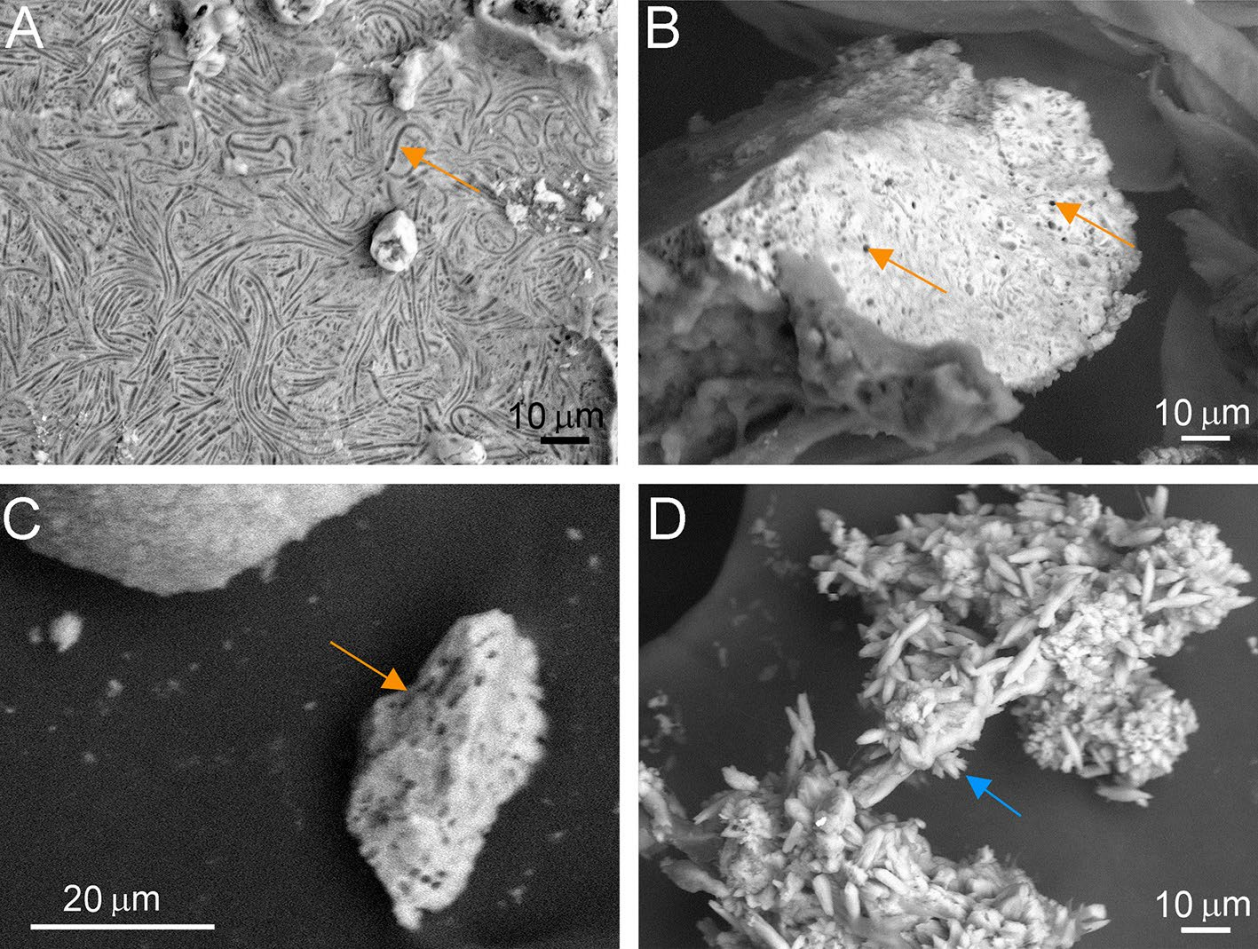
SEM images of precipitates induced by *Peribacillus simplex* OAR-202 incubated for 26 weeks using different Mg^2+^/Ca^2+^ ratios: (**A**) 0 (B4 medium); (**B**) 0.5 (B4M0.5 medium); (**C**) 1 (B4M1 medium); (**D**) 1.5 (B4M1.5 medium). Orange arrows: bacteria-shaped holes; blue arrow: needle-shaped crystals.

SEM analyses showed micron-sized holes in the form of rod-shaped bacterial cells (Fig. 5, Supplementary Fig. S3 online), which might be the consequence of the metabolic activity of the strain, i.e., the aerobic glucose oxidation resulted in weak acid production that dissolved carbonate crystals. Rod-shaped holes and tubes could also have formed as the crystals grew around the cells, which were subsequently lysed. Enyedi et al.^27^ and Sanchez-Moral et al.^30^ reported similar observations by cultivating bacteria, isolated from speleothems in Baradla Cave (Hungary, ~ 10 °C cave air temperature) and moonmilk deposits of a similar temperature in Altamira Cave (Spain). Crystal aggregates comprising rice-shaped crystals also formed among spherulitic crystals during incubation at Mg^2+^/Ca^2+^ = 1.5 (Fig. 5D). It is of interest that similar rice-shaped, inorganic calcite crystals were formed during freezing experiments in ice-rimmed pools of Eisriesenwelt, an ice cave in central Austria^63^.

**Figure 6:**
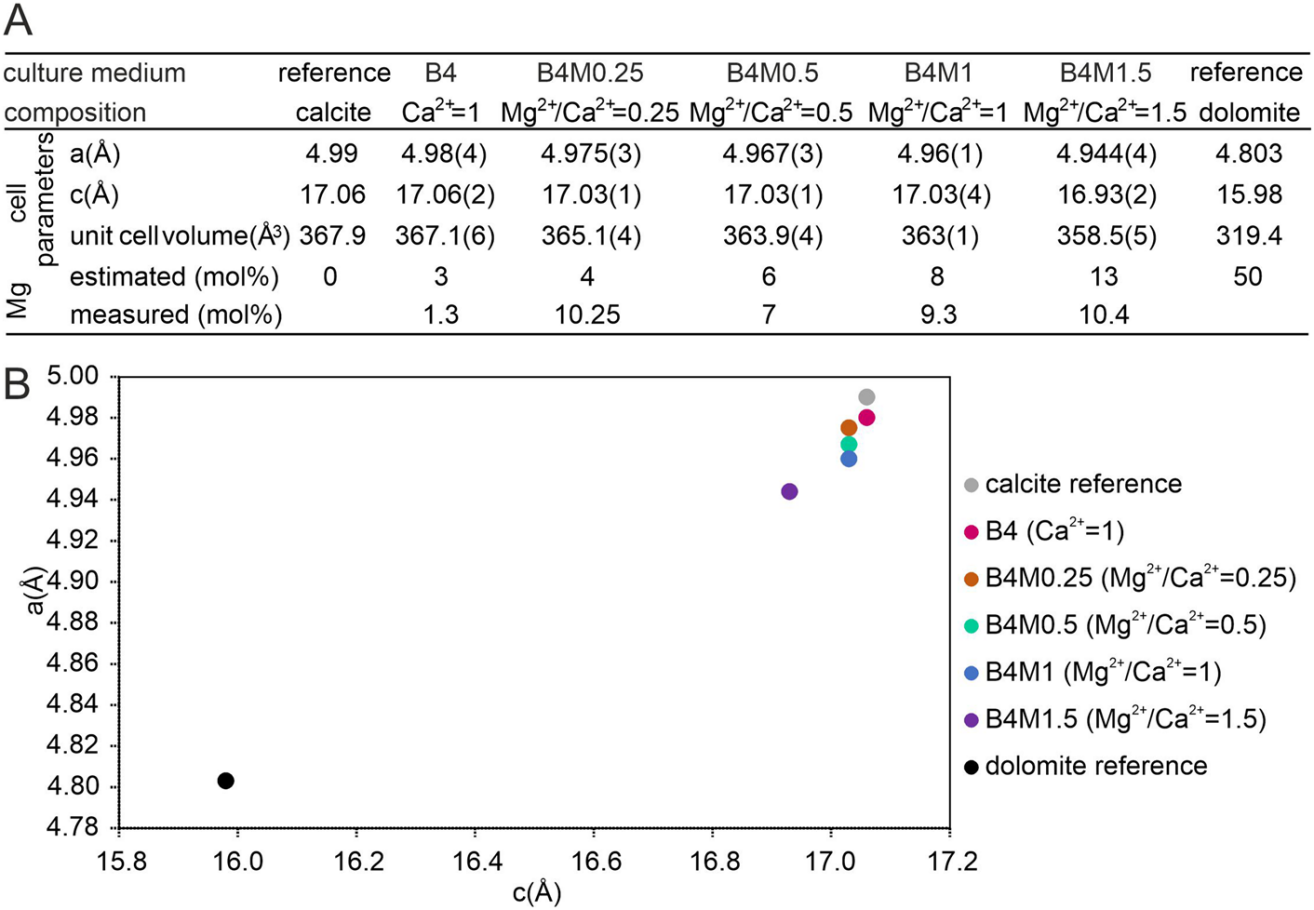
Mg^2+^/Ca^2+^ molar ratios of *Peribacillus simplex* OAR-202 carbonate precipitates cultivated on media based on cell parameters and EDS data. (**A**) Refined cell parameters of the crystals compared to pure calcite and pure dolomite, (**B**) cross plot of the *a* versus *c* parameters of the crystals.

The Mg-content of the precipitates follows the increasing Mg^2+^/Ca^2+^ ratio of the media (0; 0.25; 0.5; 1; 1.5), and up to ~ 10 mol% Mg were incorporated into the crystal structure (Fig. 6; Supplementary Fig. S4 online), consistent with energy dispersive spectroscopy (EDS) measurements (Fig. 6A; Supplementary Table S10 online). Zhang et al.^61^ also reported ~ 10 mol% Mg in calcite formed by cultivating an *Arthrobacter* sp. strain MF-2 using a medium with Mg^2+^/Ca^2+^ = 1.5. Precipitates of bacterial strains cultivated on a M2 medium with Mg^2+^/Ca^2+^ = 2 also showed an increased Mg-content (12.9–14.0 mol%)^62^ that may indicate a thermodynamic barrier for Mg incorporation in bacteria-induced Mg-calcite.

**Figure 7:**
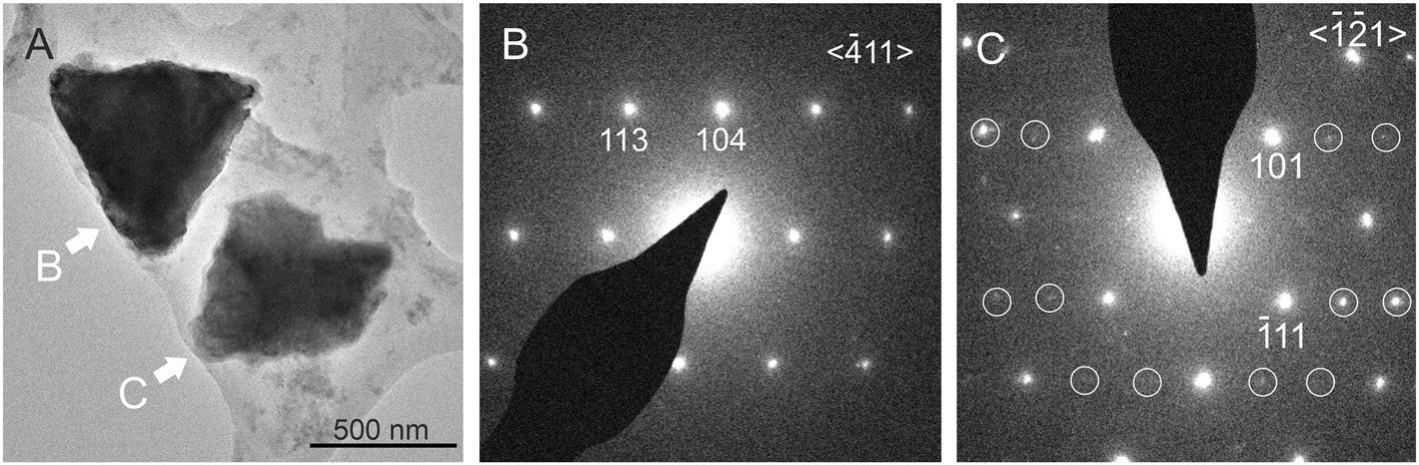
TEM images of carbonates precipitated by *Peribacillus simplex* OAR-202 on B4M1.5 medium at a molar Mg^2+^/Ca^2+^ ratio of 1.5. (**A**) Bright-field TEM image of two triangular-shaped crystals. (**B**) SAED pattern of the dark contrast crystal corresponds to calcite. (**C**) SAED pattern of the bright contrast crystal shows aragonite with satellite reflections (white circles), which can be associated with monoclinic aragonite^39^.

Although no aragonite was detected by X-ray diffraction, transmission electron microscopic (TEM) measurements (Fig. 7) of the precipitates from the strain OAR-202, identified as *Peribacillus simplex* and incubated on a medium with a Mg^2+^/Ca^2+^ ratio of 1.5 indicate the presence of small amounts of aragonite besides Mg-calcite. Both carbonate polymorphs are characterized by triangular morphologies and a similar size (~ 500 nm). Energy-dispersive X-ray analyses indicate no Mg in the aragonite and ~ 2 mol% Mg in the calcite (Supplementary Fig. S5 online). The polymorphs are separated only by an ~ 100 nm-thin EPS film, which suggests that the microenvironment of EPS plays an important role in polymorph selection. Interestingly, the selected-area electron diffraction (SAED) pattern shows aragonite satellite reflections, which are consistent with monoclinic aragonite detected in drip water samples from Obstans Ice Cave^39^. TEM observations suggest that bacteria can also precipitate this unusual aragonite form.

The results suggest that the bacterial EPS-covered surfaces of speleothems have an important role in the selection of calcium carbonate polymorphs, and that its extent depends on the amount of available biomass in this cryogenic environment. The components of the EPS matrix on the speleothems are produced by different members of the microbial community. Although the effect of only one strain on a medium containing high levels of organic matter was analyzed, the results can be generalized for microbially *induced* carbonate precipitation if the microbial metabolic processes are indeed able to increase the precipitation by alkalinization, or they produce an acidic metabolic product that is taken up by other members of the microbiota, and vice versa. The relatively high incidence of known calcium carbonate precipitating *Pseudomonas*, *Flavobacterium*, *Crossiella* in the amplicon library suggests that the capacity of the microbiota to precipitate calcium carbonate can be associated with heterotrophic ammonification, dissimilative nitrate reduction and/or autotrophic CO_2_ fixation.

## Conclusions

Ice caves provide extreme habitats (no light, near-freezing temperatures, low nutrient supply) for microbial life. Although only a small number of bacterial cells with elongated morphology were identified on the surface of speleothems from Obstans Ice Cave, a diverse bacterial taxonomic composition was detected in the most abundant phyla of Pseudomonadota, Actinomycetota, Acidobacteriota and Bacteroidota by next-generation amplicon sequencing and cultivation methods. Based on metabolic tests and literature data, most of the detected bacteria might have an aerobic chemoorgano-heterotrophic metabolism, but they also perform different processes in the nitrogen biogeochemical cycle.

Experiments demonstrate that numerous strains induce calcium carbonate precipitation at incubation temperatures of 10 °C and 21 °C, stimulated by increasing temperature. As a result of an increasing Mg^2+^/Ca^2+^ ratio of the media, Mg-free calcite is superseded by Mg-calcite containing up to ~ 10 mol% Mg. Our study shows that in addition to vaterite and amorphous calcium carbonate bacteria can also induce precipitation of aragonite in the close (100 nm) vicinity of calcite, which emphasizes that bacterial EPS plays an important role in carbonate polymorph selection.

## Methods

### Sampling

Wet 20 × 20 cm^2^ areas of the cave walls coated and uncoated by calcium carbonate were sampled using sterile cotton swabs in 7.5 ml sterile physiological saline solution. Samples obtained from surfaces of aragonite, calcite and from a cave wall lacking a carbonate coating were named OAR, OCA and OCW, respectively. Fine-grained clastic sediments were collected with sterile spoons from the cave floor as control samples (OSE1 and OSE2). 22.5 ml drip water and 18 mg of ice were collected using sterile Falcon tubes (samples ODW and OIC, respectively). All the samples were stored at 4–8 °C until they were processed in the laboratory within 12 h. The mineralogical composition of the clastic cave sediments (OSE1 and OSE2) was analyzed using a RIGAKU D/MAX RAPID II X-ray diffractometer following the method described in Kovács et al.^64^.

### Electron microscopy

Hydromagnesite and aragonite precipitates inhabited by microorganisms were investigated by SEM. The samples were fixed with 5% glutaraldehyde in 0.2 M sodium cacodylate buffer (pH 7) for 3 h. The samples were rinsed twice for 10 min in 0.1 M sodium cacodylate buffer (~ pH 7) and were frozen at −95 °C until they were placed in an Edwards freeze-dryer for 5–10 h at −60 °C operating at 2 × 10^–2^ mbar. The dried samples were sputter-coated with gold and examined using a Zeiss EVO 40 SEM at 10 kV accelerating voltage.

### Environmental DNA extraction and next-generation DNA sequencing

The microbial community DNA from the cave sediment OSE1 and OSE2 samples (0.25 g) was extracted using the Qiagen DNeasy PowerSoil Kit according to the manufacturer’s instructions with the exception that the cell disruption step was performed by shaking at 30 Hz for 2 min in a Mixer Mill MM301 (Retsch, Haan, Germany). Water, ice, and surface carbonate samples were also processed with the same kit, but they were filtered through sterile Millipore (0.22 μm pore size) polycarbonate filter membranes. Filters were cut into fragments and put into the PowerBead Tubes, and 50 µl Solution C6 was used at the elution step to concentrate DNA. DNA concentration was measured using the Qubit 2.0 (Invitrogen) and Qubit dsDNA HS Assay Kit (Thermo Fisher Scientific).

For bacterial community examination, the V3–V4 region of the 16S rRNA gene was used. B341F and B805NR universal bacterial primers^65^ as well as A519F^66^ and Arch855R^67^ archaeal primers extended with Fluidigm CS1 and CS2 adapter sequences were applied to generate amplicon libraries by polymerase chain reaction (PCR). In 20 μL final volume, 1× HF Phusion Buffer, 0.2 mM dNTPs, 0.4 μg μL^−1^ Bovine Serum Albumin, 0.5 μM of each primer, 0.4 U Phusion Hot Start II High-Fidelity DNA Polymerase (Thermo Fisher Scientific) were prepared in triplicates. The bacterial PCRs thermal conditions involved an initial denaturation at 98 °C for 3 min, which was followed by 25 cycles of denaturation at 95 °C for 10 s, annealing at 55 °C for 30 s and extension at 72 °C for 30 s. The final extension was performed at 72 °C for 5 min. For archaeal PCRs, the following thermal profile was used: initial denaturation at 98 °C for 5 min, 25 cycles of denaturation at 95 °C for 30 s, annealing at 60 °C for 30 s, extension at 72 °C for 30 s, and final extension at 72 °C for 10 min. Amplicons libraries were pooled and normalized using Qubit 2.0. They were subjected to quality control and sequenced on the Illumina MiSeq platform (Illumina Inc., San Diego, USA) at the Genomics Core, Research Technology Support Facility (Michigan State University, Trowbridge, USA) using a MiSeq Reagent Kit v2 (500 cycle).

Mothur v.1.40.5.^68^ was used for bioinformatical analyses following the MiSeq SOP manual (https://mothur.org/wiki/miseq_sop/ accessed at: 17.03.2020.)^69^. Sequences were quality-filtered by fixing deltaq to 10, minlength to 400, maxlength to 500, maxambig to 0 and maxhomop to 7 to minimize the amplification and sequencing bias. SILVA Release132 SSU NR reference database was used for sequence alignment and classification above 80 bootstrap values^70^. Chimeric and singleton sequences were removed using the UCHIME program^71^ and the process according to Kunin et al.^72^, respectively. ‘Unknown’, ‘Eukaryota’, ‘Archaea/Bacteria’, ‘Chloroplast’ and ‘Mitochondria’ labelled sequences were excluded for bacterial and archaeal results. Operational taxonomic units (OTUs) were attributed at 97% similarity threshold levels^73^. Unidentified OTUs were assigned to the closest identified taxonomical levels. The nomenclature proposed by Oren and Garrity was used^74^. Good coverage, Chao1, Shannon and Inverse Simpson indices were computed using mothur. Bubble charts were constructed using the ggplot2 package^75^ in R^76^. The sequences of the dominant (> 5% relative abundance) OTUs were compared with the BLAST database (https://blast.ncbi.nlm.nih.gov/Blast.cgi accessed at: 17.03.2020.) to identify similar sequences in previously analyzed samples.

### Cultivation, identification and characterization of bacterial strains

Ten-fold serial dilutions were prepared from OSE1, OAR, OCA and OCW samples using sterile water, and 200 µl solution was plated onto the following solid media (pH 7): R2A agar medium (containing DSMZ Medium 830 supplemented with 0.6 g/l MgSO_4_ × 7H_2_O, 0.3 g/l CaCl_2_ and 12.5 g/l gelrite instead of agar–agar), 10% R2A medium with gelrite, Casein Mineral (CM) medium^77^ and OB4M1.5 medium. The latter was the B4 medium^28^ modified by adding 0.5 g/l glucose, 2 g/l yeast extract and 5.2 g/l MgSO_4_ × 7H_2_O. These media were chosen to detect slowly growing, oligotrophic and high Ca^2+^ and Mg^2+^-tolerant bacteria. The original samples were also placed on the surface of the agar media in the form of 200 µl droplets. After 4–5 weeks of dark incubation aerobically at 10 °C, discrete bacterial colonies with different morphology were isolated, maintained and identified.

Genomic DNA from the pure cultures was extracted with sterile glass beads method as described by Enyedi et al.^78^. The 16S rRNA gene of the strains was amplified by PCR with 27F forward^79^ and 1401R reverse^80^ bacterial primers. The reaction mixture and the temperature profile of the reactions were specified in Enyedi et al.^78^. The amplified 16S rRNA gene of strains was sequenced with the Sanger-method using 27 forward primer^79^ at LGC Genomics (Berlin, Germany). Manual correction of chromatograms was performed using the Chromas software (version 2.6.6., Technelysium Pty Ltd., Australia). The closest relative type strain was attributed to every bacterial strain based on the partial 16S rRNA gene sequences using the EzBioCloud database^81^.

Biochemical tests were applied to characterize the metabolic activity of bacterial strains: Barritt’s Voges-Proskauer tests, production of hydrogen sulfide from cysteine, NH_3_ production from peptone, urease activity, and the oxidation-fermentation test of D-glucose. The tests were performed in duplicates according to the procedures described by^28^. Results were compared based on the intensity of the color changes and/or the production of precipitation in the case of NH_3_ detection.

### Microbially induced carbonate precipitation experiments

The aim of these experiments was to examine the calcium and magnesium carbonate precipitation capacity of taxonomically diverse bacterial strains. Precipitation capacity was also tested for several different strains of the same species to investigate possible strain-specific differences. For 23 strains we applied solid Ca-containing B4 and equal concentrations (14.2 mM) of Mg^2+^ and Ca^2+^ containing B4M1 media and adjusted the pH to 7.5 using 0.1 N NaOH (Supplementary Table S11 online). The surface of 9 × 9 cm solid agar plates was inoculated with two cross-shaped (diameter ~ 2 cm) identical bacterial strains. The Petri dishes were sealed with Parafilm to prevent dehydration at 21 °C^28^ but allowed diffusion of gases such as oxygen and carbon dioxide. At 10 °C, four Petri dishes in a batch were placed in aluminum foil to prevent dehydration and contamination of the medium with other bacteria or mold from the air. The bacterial strains and the inoculated negative control samples were incubated together in the same 10 °C refrigerator for 26 weeks and in the same 21 °C room for 52 weeks to provide identical environments for the experiments in terms of humidity and partial CO_2_ pressure. The Petri dishes were left intact for the duration of the experiment and no components were added to the medium.

Stereomicroscope images were taken weekly of the cultures at 40 × magnification using a Canon camera attached to the microscope and QuickPHOTO CAMERA 3.1 software to follow the carbonate precipitation process according to the procedure described by Lange-Enyedi et al.^28^. The images were analyzed using NIS-Elements BR Analysis 5.21.02. software (Nikon) to measure the percentage of crystal coverage on the colonies’ surface. No precipitation occurred on negative control plates. After 26 weeks, measurable amounts (0.5–1 mg) of mineral precipitates were collected from one or many colonies of bacterial strains. Precipitates of each crystal morphology group were examined for their mineralogical composition with micro-X-ray diffraction (micro-XRD) following the protocol described in the section “Sampling”.

The acidic-basic characteristics of B4M1 plates were measured during the carbonate precipitating experiments using a cresol-red pH indicator (B4M1-CR) following the methodology described for the B4 medium^28^. The experiments were monitored for 134 days on B4M1-CR medium at 21 °C and 10 °C incubation temperatures.

To observe the effect of different Mg^2+^/Ca^2+^ ratios (0, 0.25, 0.5, 1, 1.5) at pH 7.5, different precipitating media were prepared (Supplementary Table S11 online) by adding increasing amounts of MgSO_4_ × 7H_2_O (0, 3.55, 7,1, 14.2, 21.3 mM) to the same amount of calcium-acetate (14.2 mM) (Supplementary Table S11 online). The media were incubated with *Peribacillus simplex* OAR-202, a bacterial strain tested positive for calcium and magnesium carbonate precipitation. Based on the consistent mineralogical results of the experiments at 21 °C and 10 °C, the incubation temperature was set at 21 °C. After 26 weeks, mineral precipitates in the colonies from every cultivation medium were examined for their mineralogical composition using the micro-XRD diffractometer. The unit-cell parameters of calcite precipitates of *Peribacillus simplex* strain OAR-202 were refined based on the 104, 110, 113, 018 and 116 reflections using the UnitCell software^82^. The Mg-content of the calcite precipitates was estimated from these refined cell parameters following the procedure by Goldsmith et al.^83^. The morphology, texture, and chemical composition of the precipitated crystals in contact with the bacterial strains and EPS were investigated in low vacuum mode (100 Pa) with a JEOL JSM-IT700HR SEM equipped with an EDS operating at 20 kV. The Mg-content was obtained based on three EDS measurements of atomic-% Mg compared to atomic-% Ca.

The precipitates of *Peribacillus simplex* strain OAR-202 incubated on B4M1.5 (Mg^2+^/Ca^2+^ = 1.5, similarly to the Obstans Ice Cave drip water) were also examined using a 200 keV Talos Thermo Scientific TEM. Samples were prepared following the procedure described by Enyedi et al.^27^, and bright-field TEM images were obtained as well as SAED patterns.

### Supplementary Information

Supplementary Information.

## Data Availability

Raw next-generation amplicon sequence data can be accessed under the NCBI BioProject ID PRJNA992252. The 16S rRNA gene sequences of the strains were deposited into the GenBank database under the following accession numbers: OR253331-OR253440.
