# Supplementary material for: Calcium carbonate precipitating extremophilic bacteria in an Alpine ice cave
Source: Sci Rep. 2024 Feb 1;14:2710. doi: 10.1038/s41598-024-53131-y (PMC10834452; doi:10.1038/s41598-024-53131-y)
Supplement: Supplementary file 1 — Supplementary Information. [file 41598_2024_53131_MOESM1_ESM.docx]

**Supplementary material**

**Calcium carbonate precipitating extremophilic bacteria in an Alpine ice cave**

Nóra Tünde Lange-Enyedi^1,2^, Péter Németh^1,3*^, Andrea K. Borsodi^2,4^, Christoph Spötl^5^, Judit Makk^2^

^1^Institute for Geological and Geochemical Research, HUN-REN Research Centre for Astronomy and Earth Sciences, Budaörsi út 45, H-1112 Budapest, Hungary

^2^Department of Microbiology, Institute of Biology, Faculty of Science, ELTE Eötvös Loránd University, Pázmány P. sétány 1/C, H-1117 Budapest, Hungary

^3^Research Institute of Biomolecular and Chemical Engineering, Nanolab, University of Pannonia, Egyetem út 10, H-8200, Veszprém, Hungary

^4^Institute of Aquatic Ecology, HUN-REN Centre for Ecological Research, Karolina út 29, H-1113 Budapest, Hungary

^5^Institute of Geology, University of Innsbruck, Innrain 52, A-6020 Innsbruck, Austria

*Correspondence to: nemeth.peter@csfk.org


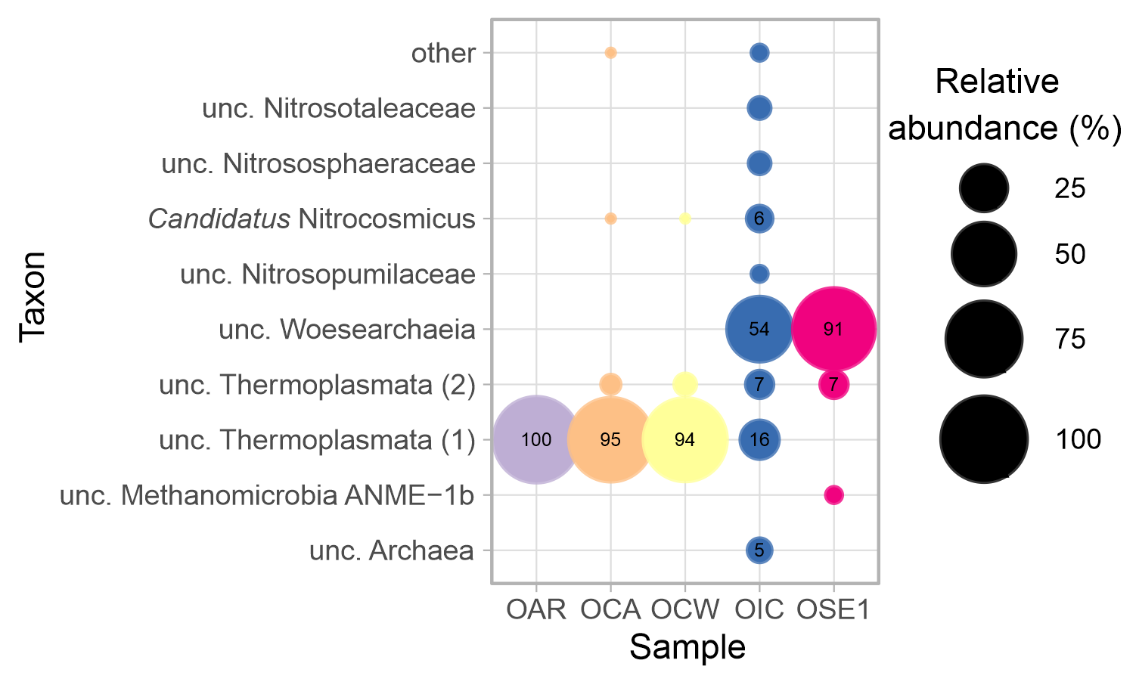


Figure S1. Distribution of archaeal OTUs among genera, based on the amplicon sequencing of V3-V4 regions of 16S rRNA gene sequences, of Obstans Ice Cave (OAR: Obstans aragonite; OCA: Obstans calcite; OCW: Obstans cave wall; OIC: Obstans ice; OSE1: Obstans sediment; unc.: uncultured).


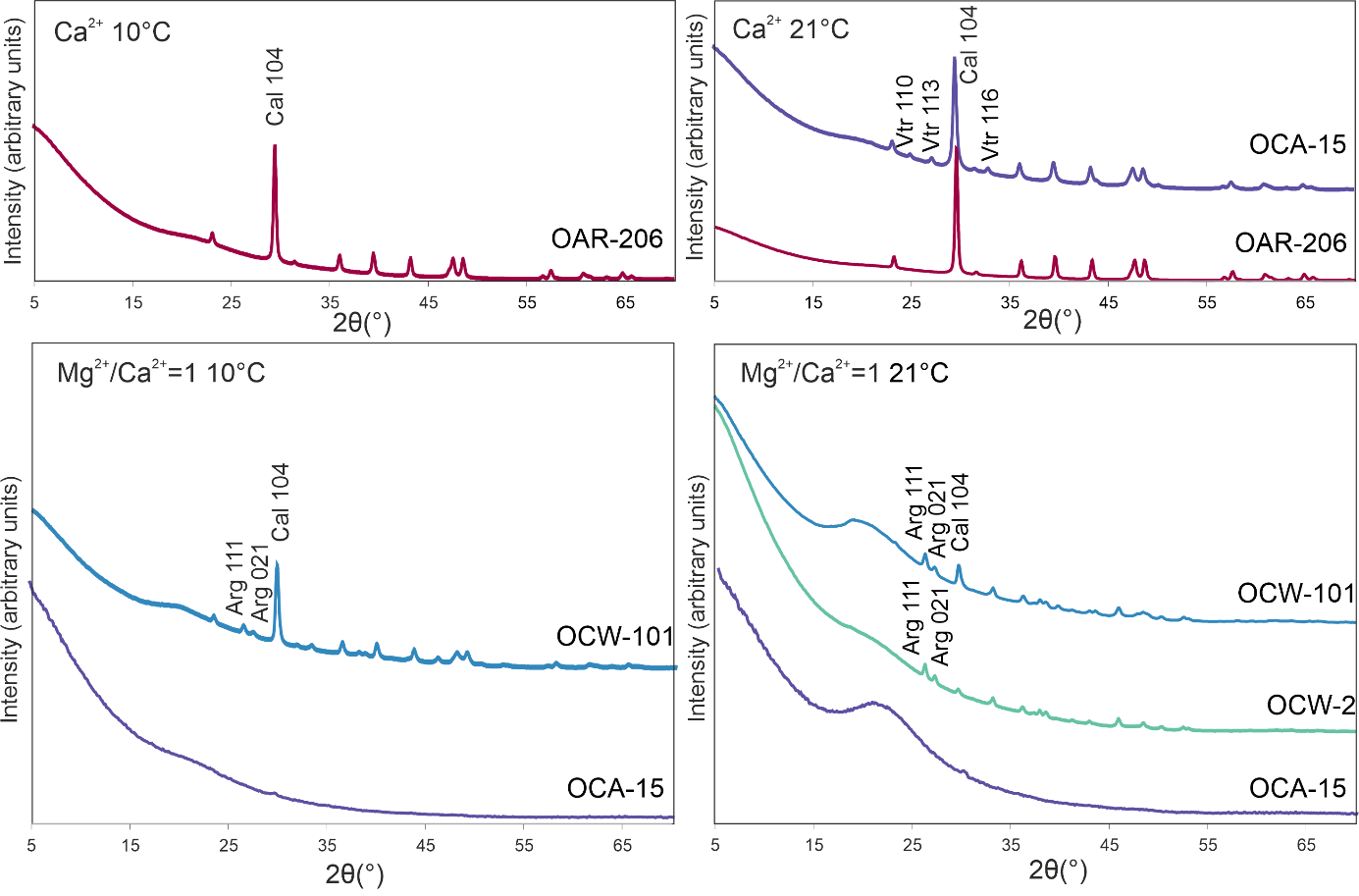


Figure S2. Diffractograms and the most intense peaks of the carbonate precipitates isolated from selected bacterial colonies, incubated at 10 and 21 °C and at Mg^2+^-free and Mg^2+^/Ca^2+^ = 1 conditions for 26 weeks (Arg: aragonite, Vtr: vaterite, Cal: calcite).


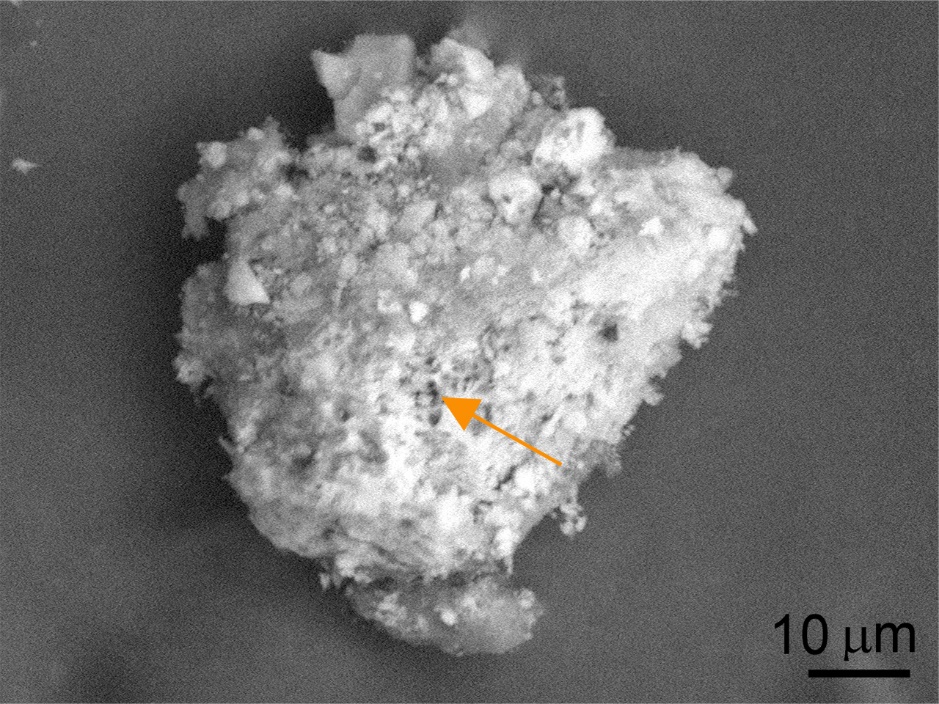


Figure S3. Carbonate aggregate precipitated by *Peribacillus simplex* OAR-202 that was incubated for 26 weeks at Mg^2+^/Ca^2+^= 0.25. Orange arrow points to bacteria-shaped hole.


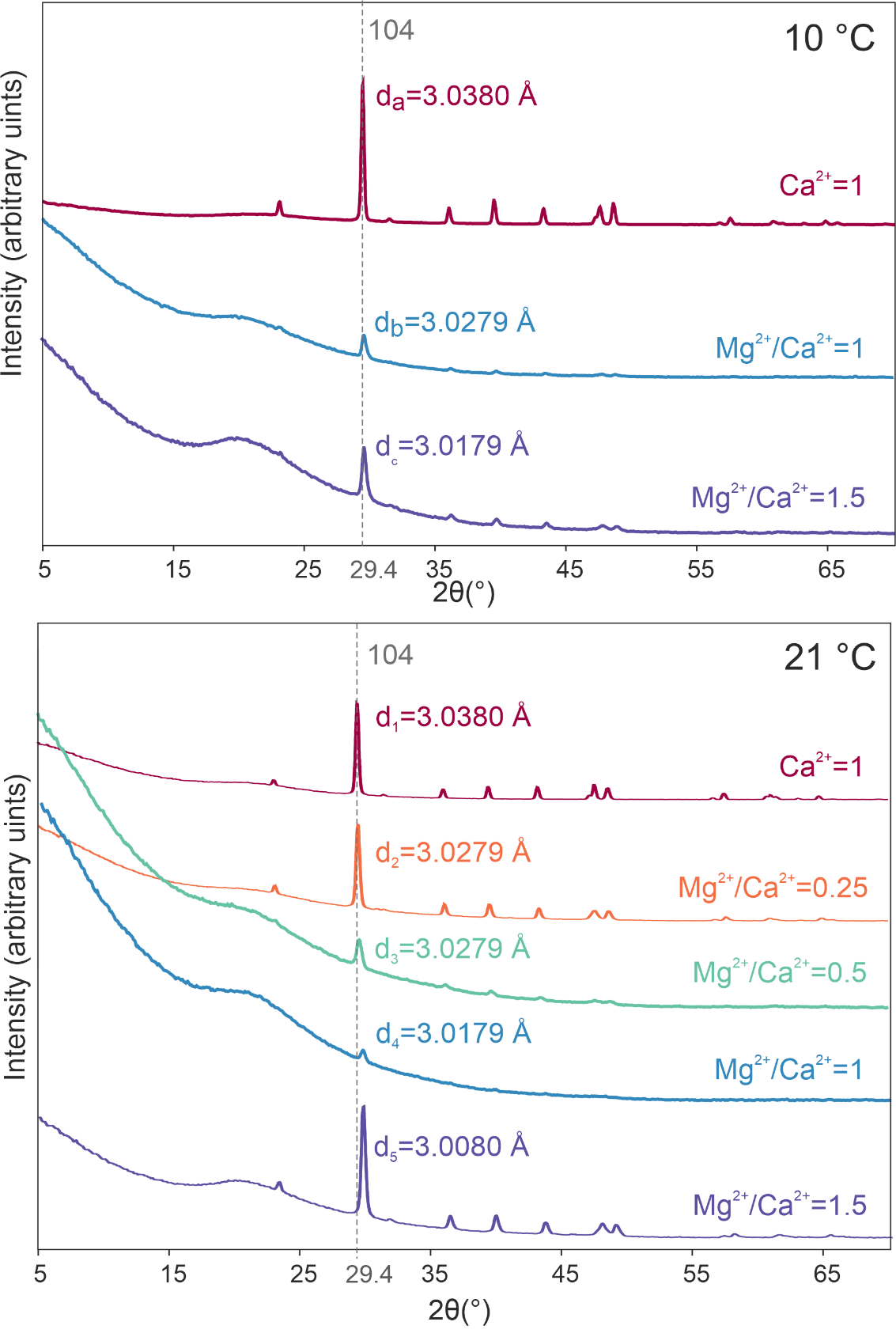


Figure S4. Diffractograms and the *104* calcite peak shift of the precipitates, isolated from bacterial colonies of *Peribacillus simplex* strain OAR-202 incubated at different temperatures and Mg^2+^/Ca^2+^ ratios for 26 weeks.


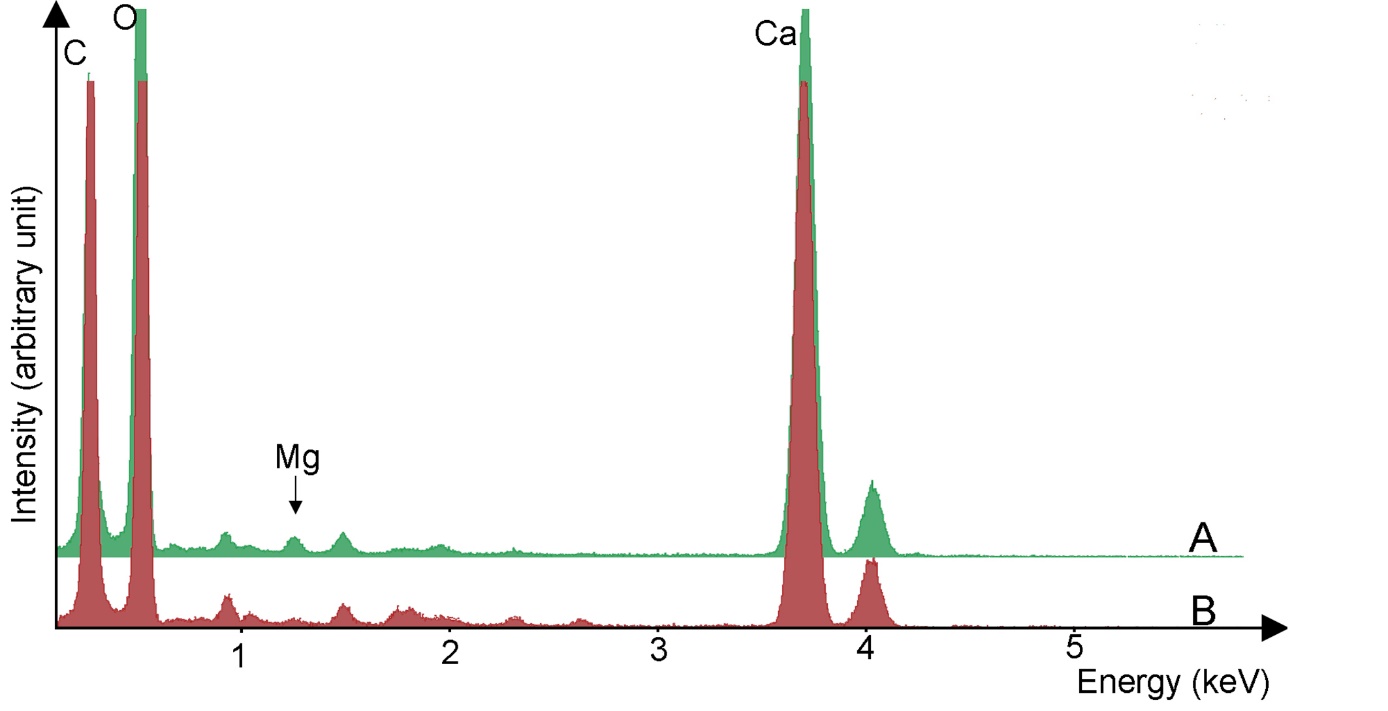


Figure S5. TEM EDS analysis of the carbonate precipitates, formed by *Peribacillus simplex* OAR-202 on B4M1.5 medium with a molar Mg^2+^/Ca^2+^ ratio of 1.5. A) Crystal B on Figure 7 contains ~2 mol% Mg. B) Crystal C on Figure 7 contains no detectable Mg.

Table S1. Relative abundance of identified bacterial sequences at the phylum and the genus level in Obstans Ice Cave (genera >1% relative abundance in a sample are in bold. Abbreviations: ODW, Obstans drip water; OIC: Obstans ice; OAR: Obstans aragonite; OCA: Obstans calcite; OCW: Obstans cave wall; and OSE1-2: Obstans sediment, unc.: uncultured).

| **Taxon** | **ODW** | **OIC** | **OAR** | | **OCA** | **OCW** | **OSE1** | **OSE2** |
| --- | --- | --- | --- | --- | --- | --- | --- | --- |
| **Acidobacteriota** | **1.25** | 0.31 | **20.27** | | **12.81** | **6.29** | **9.77** | 0.08 |
| unc. Acidobacteria |  |  |  | | 0.01 |  |  |  |
| Acidipila | 0.02 |  |  | |  |  |  |  |
| unc. Acidobacteriaceae (Subgroup 1) | 0.01 |  |  | |  |  |  |  |
| unc. Acidobacteriales |  | 0.01 |  | |  |  |  | 0.01 |
| Bryobacter | 0.04 |  | 0.09 | | 0.01 | 0.17 | 0.07 |  |
| Candidatus Solibacter | 0.01 |  |  | |  |  |  |  |
| Paludibaculum | 0.01 | 0.01 | 0.02 | | 0.01 | 0.01 | 0.33 |  |
| unc. Solibacteraceae (Subgroup 3) | 0.04 |  | 0.01 | | 0.01 | 0.04 | 0.12 |  |
| unc. Subgroup 2 | 0.03 |  |  | |  |  |  |  |
| Aridibacter | 0.02 |  |  | | 0.01 |  |  |  |
| **Blastocatella** | 0.07 |  | **8.82** | | **5.57** | **3.46** | 0.04 |  |
| unc. Blastocatellaceae |  |  |  | | 0.01 |  | 0.04 |  |
| **unc. Blastocatellaceae** | 0.24 | 0.21 | 0.75 | | **3.21** | **1.09** | **3.50** |  |
| unc. JGI 0001001-H03 | 0.10 |  | 0.16 | | 0.11 | 0.39 | 0.90 |  |
| unc. Blastocatellaceae | 0.01 |  |  | |  |  |  |  |
| unc. Blastocatellia (Subgroup 4) |  |  |  | | 0.08 | 0.01 |  |  |
| unc. Pyrinomonadaceae |  |  |  | | 0.02 |  |  |  |
| **unc. RB41** | 0.23 | 0.02 | **10.08** | | **3.61** | 0.69 | **3.97** |  |
| unc. Subgroup 7 | 0.03 | 0.02 | 0.09 | | 0.04 | 0.24 | 0.59 |  |
| unc. Subgroup 17 | 0.04 |  |  | | 0.01 | 0.01 | 0.03 |  |
| unc. Subgroup 22 | 0.05 |  |  | |  |  | 0.04 |  |
| unc. Subgroup 6 | 0.27 | 0.03 | 0.21 | | 0.13 | 0.14 | 0.09 |  |
| unc. Subgroup 6 |  | 0.01 | 0.04 | |  |  | 0.06 |  |
| Luteitalea |  |  |  | |  | 0.04 | 0.01 | 0.08 |
| unc. Subgroup 6 Family |  |  |  | |  |  |  |  |
| unc. Subgroup 10 | 0.01 |  |  | |  |  |  |  |
| **Actinomycetota** | **11.40** | **8.53** | **35.33** | | **27.97** | **22.99** | **16.93** | **29.08** |
| **unc. 0319-7L14** | 0.11 |  | 0.01 | | 0.01 | 0.06 | 0.13 | **1.06** |
| unc. Acidimicrobiia | 0.01 |  |  | |  |  |  |  |
| **unc. Acidimicrobiia** | 0.04 |  | 0.11 | | **1.45** | 0.79 | 0.28 | 0.09 |
| unc. Actinomarinales |  |  |  | |  |  |  |  |
| unc. Actinomarinales |  |  |  | |  |  | 0.02 | 0.25 |
| unc. Actinomarinales | 0.05 |  |  | |  |  | 0.23 |  |
| unc. IMCC26256 | 0.34 |  |  | |  | 0.09 | 0.02 | 0.17 |
| Iamia | 0.02 | 0.02 | 0.19 | | 0.04 | 0.14 | 0.09 | 0.01 |
| unc. CL500-29 marine group | 0.01 |  | 0.06 | | 0.02 | 0.03 | 0.08 |  |
| unc. Ilumatobacteraceae |  |  |  | |  |  | 0.01 |  |
| unc. Ilumatobacteraceae | 0.26 | 0.04 | 0.88 | | 0.51 | 0.91 | 0.19 | 0.67 |
| **unc. Microtrichales** | 0.01 |  | 0.46 | | **1.14** | 0.52 | 0.15 | 0.36 |
| unc. Microtrichales |  |  |  | |  |  | 0.02 |  |
| **unc. Acidimicrobiia** | 0.20 | 0.04 | **1.52** | | **3.09** | 0.72 | 0.42 | **11.92** |
| **unc. Actinobacteria** | 0.35 | 0.15 | | **1.80** | **1.61** | 0.83 | **1.96** | 0.50 |
| Corynebacterium | 0.03 |  | |  |  | 0.01 |  |  |
| Lawsonella |  |  | |  |  |  |  |  |
| Tomitella | 0.03 |  | |  |  | 0.03 |  |  |
| Dietzia | 0.04 |  | |  |  |  |  |  |
| Mycobacterium | 0.01 |  | |  |  |  |  |  |
| Nocardia | 0.01 |  | | 0.10 |  | 0.01 |  |  |
| Rhodococcus | 0.13 |  | |  |  | 0.04 |  |  |
| Acidothermus |  |  | |  |  | 0.02 |  |  |
| Jatrophihabitans | 0.02 |  | |  |  |  |  |  |
| Frankiales |  | 0.01 | | 0.01 |  | 0.01 |  | 0.01 |
| Blastococcus | 0.08 |  | |  |  |  |  |  |
| Sporichthya |  |  | | 0.24 | 0.59 | 0.05 |  |  |
| **Taxon**  Table S1. (continued) | **ODW** | **OIC** | | **OAR** | **OCA** | **OCW** | **OSE1** | **OSE2** |
| unc. Sporichthyaceae |  |  | | 0.13 | 0.01 | 0.03 | 0.01 |  |
| **unc. Frankiales** | 0.11 | 0.48 | | **1.92** | 0.72 | **1.34** | 0.21 | 0.30 |
| Quadrisphaera | 0.01 |  | | 0.02 |  |  |  | 0.02 |
| Brevibacterium | 0.02 |  | |  |  | 0.01 |  |  |
| unc. Cellulomonadaceae |  |  | |  |  | 0.01 |  |  |
| Brachybacterium | 0.23 | 0.25 | |  |  | 0.22 |  |  |
| unc. Dermabacteraceae |  | 0.02 | |  |  |  |  |  |
| Kytococcus |  |  | |  |  | 0.01 |  |  |
| unc. Intrasporangiaceae | 0.21 | 0.01 | |  |  | 0.07 |  |  |
| Ornithinimicrobium |  |  | |  |  | 0.03 |  |  |
| Oryzihumus |  | 0.02 | |  |  |  |  |  |
| Cryobacterium |  | 0.42 | |  |  |  |  |  |
| unc. Microbacteriaceae | 0.02 | 0.04 | |  |  | 0.09 |  |  |
| Microbacterium |  | 0.01 | |  |  |  |  |  |
| Acaricomes |  | 0.08 | |  |  |  |  |  |
| **Arthrobacter** | 0.12 | 0.03 | |  |  | **3.11** |  |  |
| Citricoccus | 0.06 |  | |  |  | 0.01 |  |  |
| Kocuria | 0.03 | 0.01 | |  |  | 0.05 |  |  |
| **unc. Micrococcaceae** | 0.21 | **4.79** | |  | 0.01 | 0.46 | 0.05 |  |
| **Micrococcus** | **2.81** | **1.19** | |  |  | **3.31** |  |  |
| Rothia |  |  | |  |  | 0.02 |  |  |
| unc. Micrococcales |  | 0.10 | |  |  | 0.04 |  |  |
| Sanguibacter | 0.07 |  | |  |  | 0.01 |  |  |
| unc. Micromonosporaceae |  |  | | 0.02 |  |  | 0.01 | 0.01 |
| Aeromicrobium | 0.02 | 0.02 | |  | 0.02 | 0.01 |  |  |
| Kribbella | 0.06 |  | |  | 0.03 | 0.03 |  |  |
| Marmoricola | 0.06 | 0.01 | |  | 0.01 | 0.04 | 0.10 |  |
| unc. Nocardioidaceae | 0.22 | 0.02 | | 0.24 | 0.24 | 0.88 | 0.01 | 0.01 |
| Nocardioides | 0.20 | 0.30 | |  | 0.71 | 0.98 |  |  |
| Cutibacterium | 0.02 |  | |  |  |  |  |  |
| unc. Propionibacteriaceae |  |  | |  |  | 0.01 |  |  |
| Propionicimonas | 0.01 |  | |  |  |  |  |  |
| unc. Propionibacteriales |  |  | |  | 0.01 |  |  |  |
| Actinophytocola |  |  | | 0.03 |  |  |  |  |
| Amycolatopsis |  |  | | 0.07 |  | 0.03 | 0.10 | 0.01 |
| **Crossiella** | **1.61** |  | | 0.57 | 0.13 | **1.14** | **9.56** | 0.42 |
| **Pseudonocardia** | 0.56 | 0.05 | | **12.51** | **4.57** | **1.68** | 0.48 | **1.97** |
| unc. Pseudonocardiaceae | 0.11 |  | | 0.02 | 0.06 | 0.07 |  | 0.01 |
| unc. Streptomycetaceae |  |  | | 0.02 |  |  | 0.01 |  |
| unc. Streptosporangiaceae |  |  | | 0.01 |  |  | 0.01 |  |
| unc. Actinobacteria | 0.06 | 0.01 | | 0.07 | 0.11 | 0.05 | 0.02 | 0.02 |
| unc. MB-A2-108 | 0.04 |  | |  |  | 0.04 |  |  |
| Euzebya | 0.02 |  | | 0.04 | 0.08 | 0.03 | 0.01 | 0.54 |
| unc. Euzebyaceae | 0.07 |  | | 0.18 | 0.01 | 0.01 |  | 0.05 |
| **unc. Euzebyaceae** | 0.08 |  | | 0.02 |  |  | 0.05 | **6.95** |
| unc. Nitriliruptoraceae |  |  | | 0.01 |  |  |  | 0.03 |
| **Rubrobacter** | 0.35 | 0.13 | | **1.41** | **3.24** | **1.62** |  |  |
| **Gaiella** | 0.25 | 0.02 | | **1.44** | 0.07 | 0.24 | **1.11** | 0.54 |
| unc. Gaiellales | 0.01 |  | |  |  |  | 0.01 |  |
| unc. Gaiellales | 0.05 |  | | 0.61 | 0.61 | 0.06 | 0.01 | 0.06 |
| **unc. 67-14** | 0.40 | 0.05 | | **5.97** | **1.21** | 0.61 | 0.55 | **1.35** |
| **Conexibacter** | 0.16 | 0.05 | | **2.34** | **5.85** | **1.07** | 0.08 | 0.79 |
| unc. JCM 18997 |  | 0.01 | | 0.01 | 0.11 | 0.01 |  |  |
| Solirubrobacter | 0.21 |  | | 0.20 | 0.07 | 0.37 | 0.02 |  |
| **unc. Solirubrobacteraceae** | 0.08 | 0.01 | | 0.37 | **1.21** | 0.30 | 0.04 | 0.11 |
| unc. Solirubrobacteraceae | 0.63 | 0.09 | | 0.28 | 0.35 | 0.45 | 0.56 | 0.10 |
| **unc. Solirubrobacterales** | 0.45 | 0.02 | | **1.44** | 0.09 | 0.19 | 0.28 | 0.76 |
| unc. Thermoleophilia |  | 0.02 | | 0.02 | 0.01 | 0.01 | 0.01 |  |
| unc. Thermoleophilia |  |  | |  |  |  | 0.04 |  |
| Armatimonadota | 0.01 |  | | 0.04 | 0.02 | 0.02 | 0.86 | 0.01 |
| **Taxon**  Table S1. (continued) | **ODW** | **OIC** | | **OAR** | **OCA** | **OCW** | **OSE1** | **OSE2** |
| unc. Armatimonadales |  |  | |  |  |  |  | 0.01 |
| unc. Fimbriimonadaceae | 0.01 |  | | 0.01 | 0.02 |  | 0.58 |  |
| unc. Fimbriimonadaceae |  |  | |  |  |  | 0.01 |  |
| unc. Armatimonadota |  |  | | 0.03 |  | 0.02 | 0.27 |  |
| Sumerlaeota (BRC1) | 0.02 | 0.03 | | 0.01 | 0.08 | 0.08 | 0.05 |  |
| unc. BRC1 | 0.02 | 0.03 | | 0.01 | 0.08 | 0.08 | 0.05 |  |
| **Uncultured Bacteria** | **2.63** | 0.39 | | 0.08 | 0.86 | 0.53 | **2.03** | 0.19 |
| **unc**. **Bacteria** | **2.63** | 0.39 | | 0.08 | 0.86 | 0.53 | **2.03** | 0.19 |
| **Bacteroidota** | **3.61** | **34.39** | | **2.63** | **10.29** | **9.42** | **6.44** | **6.54** |
| unc. Bacteroidia | 0.06 | 0.04 | |  | 0.03 |  | 0.02 |  |
| unc. Chitinophagaceae | 0.15 | 0.03 | | 0.20 | 0.91 | 0.38 | 0.35 | 0.01 |
| Ferruginibacter | 0.02 | 0.09 | |  |  | 0.02 |  |  |
| Flavihumibacter |  |  | |  |  |  |  |  |
| **Flavisolibacter** | 0.05 | 0.09 | | **2.01** | **2.46** | **1.09** | **4.22** |  |
| Flavitalea | 0.01 |  | |  |  | 0.02 |  | 0.01 |
| Lacibacter |  | 0.01 | |  |  |  |  |  |
| Niveitalea |  |  | |  |  |  | 0.01 |  |
| Parafilimonas |  |  | |  |  |  |  | 0.01 |
| Sediminibacterium | 0.01 | 0.03 | |  |  |  |  |  |
| Terrimonas |  |  | |  |  | 0.01 |  |  |
| unc. Chitinophagaceae | 0.03 | 0.02 | |  | 0.02 | 0.01 |  |  |
| unc. Chitinophagales |  |  | |  | 0.01 |  |  |  |
| unc. Saprospiraceae | 0.02 | 0.01 | |  |  |  | 0.19 |  |
| unc. Chitinophagales |  |  | |  | 0.15 |  | 0.11 |  |
| Algoriphagus | 0.03 |  | |  |  |  |  |  |
| **unc. Cyclobacteriaceae** | 0.04 |  | | 0.15 |  | 0.01 | 0.63 | **4.98** |
| **Cytophaga** | **1.01** | 0.03 | |  |  |  |  |  |
| Sporocytophaga | 0.10 |  | | 0.07 | 0.37 | 0.15 |  |  |
| unc. Cytophagales |  |  | |  | 0.01 | 0.01 |  | 0.84 |
| Hymenobacter | 0.03 | 0.01 | |  |  |  |  |  |
| Chryseolinea |  |  | | 0.01 |  |  |  |  |
| unc. Microscillaceae |  | 0.01 | |  | 0.02 | 0.01 |  |  |
| Ohtaekwangia | 0.02 |  | |  |  |  |  |  |
| unc. Microscillaceae | 0.09 | 0.01 | |  |  |  | 0.05 | 0.69 |
| Spirosoma | 0.07 |  | |  |  |  |  |  |
| unc. Spirosomaceae | 0.01 | 0.01 | |  |  | 0.03 |  |  |
| Fluviicola | 0.05 | 0.35 | |  | 0.11 |  |  |  |
| unc. Flavobacteriaceae | 0.07 | 0.27 | |  |  |  |  |  |
| **Flavobacterium** | 0.55 | **31.70** | |  | 0.01 | 0.08 |  |  |
| Gillisia | 0.01 |  | |  |  |  |  |  |
| unc. Flavobacteriales |  |  | |  | 0.01 | 0.01 |  |  |
| Chryseobacterium | 0.03 |  | | 0.01 |  |  |  |  |
| unc. AKYH767 | 0.24 |  | |  | 0.05 | 0.48 |  |  |
| unc. FFCH9454 |  |  | |  | 0.02 | 0.08 |  |  |
| unc. KD3-93 | 0.12 |  | |  | 0.83 | 0.13 |  |  |
| unc. LiUU-11-161 |  | 0.01 | |  |  |  |  |  |
| **Pedobacter** | 0.16 | 0.91 | |  | **2.93** | 0.19 |  |  |
| **unc. Sphingobacteriaceae** | 0.09 | 0.27 | | 0.02 | 0.11 | **4.52** | 0.13 |  |
| Sphingobacterium | 0.02 |  | |  |  | 0.02 |  |  |
| unc. Sphingobacteriales | 0.26 | 0.19 | |  | 0.13 | 0.15 | 0.29 |  |
| **unc. env.OPS 17** | 0.12 | 0.26 | | 0.15 | **2.07** | **1.95** | 0.03 |  |
| unc. PHOS-HE36 |  |  | |  |  |  | 0.01 |  |
| unc. BSV26 | 0.01 |  | |  |  | 0.01 |  |  |
| unc. OPB56 | 0.01 | 0.03 | |  | 0.02 |  | 0.35 |  |
| unc. SJA-28 | 0.09 |  | |  |  | 0.03 |  |  |
| unc. Rhodothermaceae |  |  | |  |  |  | 0.05 |  |
| unc. Rhodothermaceae |  |  | |  | 0.04 | 0.05 |  |  |
| Chlamydiota | 0.18 |  | |  | 0.03 | 0.03 | 0.01 |  |
| unc. Chlamydiales | 0.09 |  | |  |  |  |  |  |
| Candidatus Protochlamydia |  |  | |  |  | 0.01 |  |  |
| **Taxon**  Table S1. (continued) | **ODW** | **OIC** | | **OAR** | **OCA** | **OCW** | **OSE1** | **OSE2** |
| Neochlamydia |  |  | |  |  | 0.01 |  |  |
| unc. Parachlamydiaceae |  |  | |  | 0.03 | 0.01 | 0.01 |  |
| Candidatus Rhabdochlamydia | 0.01 |  | |  |  |  |  |  |
| unc. Simkaniaceae | 0.03 |  | |  |  |  |  |  |
| unc. cvE6 | 0.05 |  | |  |  |  |  |  |
| **Chloroflexota** | 0.72 | 0.07 | | **5.75** | **2.48** | **1.22** | **2.29** | **5.86** |
| unc. AD3 | 0.01 |  | |  |  |  |  |  |
| unc. Anaerolineae |  |  | |  | 0.01 |  |  |  |
| unc. UTCFX1 |  |  | |  |  | 0.01 |  |  |
| unc. Ardenticatenaceae |  |  | | 0.02 |  |  | 0.03 |  |
| unc. Caldilineaceae |  |  | |  |  |  | 0.24 |  |
| unc. Caldilineaceae | 0.03 |  | |  | 0.01 | 0.01 | 0.01 | 0.22 |
| unc. A4b | 0.01 |  | |  |  |  |  |  |
| unc. SBR1031 |  |  | |  |  |  | 0.01 |  |
| unc. Chloroflexi | 0.02 |  | | 0.01 |  | 0.01 |  |  |
| unc. Chloroflexia |  |  | |  |  |  |  | 0.01 |
| **unc. AKIW781** | 0.03 |  | | **1.23** | 0.27 | 0.19 | 0.04 | 0.01 |
| unc. AKYG1722 | 0.03 | 0.01 | | 0.09 | 0.02 | 0.10 | 0.13 | 0.50 |
| **unc. JG30-KF-CM45** | 0.15 | 0.04 | | **1.48** | **1.60** | 0.60 | 0.05 | 0.51 |
| unc. Thermomicrobiales |  |  | |  | 0.01 |  |  |  |
| unc. Dehalococcoidia |  |  | |  |  | 0.03 |  |  |
| unc. S085 | 0.02 |  | |  |  | 0.03 | 0.11 |  |
| unc. SAR202 clade | 0.01 |  | |  |  |  |  |  |
| **unc. Gitt-GS-136** | 0.16 |  | | 0.03 | 0.03 | 0.06 | 0.33 | **1.34** |
| **unc. JG30-KF-CM66** | 0.02 |  | | **2.35** | 0.23 | 0.03 | 0.03 |  |
| **unc. KD4-96** | 0.12 | 0.02 | | 0.46 | 0.32 | 0.09 | 0.32 | **3.27** |
| unc. C0119 | 0.02 |  | | 0.06 |  | 0.01 | 0.40 |  |
| unc. Ktedonobacteraceae | 0.03 |  | |  |  |  |  |  |
| unc. OLB14 |  |  | |  |  |  | 0.22 |  |
| unc. P2-11E |  |  | |  |  | 0.03 | 0.34 |  |
| unc. SHA-26 | 0.01 |  | |  |  | 0.01 |  |  |
| unc. TK10 | 0.05 |  | | 0.02 |  | 0.03 | 0.04 |  |
| Cyanobacteria | 0.31 | 0.04 | | 0.17 | 0.26 | 0.06 |  |  |
| unc. Caenarcaniphilales | 0.15 |  | |  |  |  |  |  |
| unc. Obscuribacterales | 0.03 |  | |  | 0.04 | 0.01 |  |  |
| unc. Vampirovibrionales |  |  | | 0.08 | 0.13 |  |  |  |
| unc. Nostocales | 0.02 |  | |  |  |  |  |  |
| unc. Leptolyngbya RV74 | 0.08 |  | |  |  |  |  |  |
| unc. Sericytochromatia | 0.02 | 0.04 | | 0.09 | 0.09 | 0.05 |  |  |
| Deinococcota | 0.18 |  | |  | 0.01 |  | 0.04 |  |
| unc. Deinococcaceae |  |  | |  |  |  | 0.04 |  |
| Deinococcus | 0.18 |  | |  |  |  |  |  |
| Meiothermus |  |  | |  | 0.01 |  |  |  |
| Candidatus Dependentiae | 0.15 | 0.01 | |  |  |  |  |  |
| unc. Babeliales | 0.09 |  | |  |  |  |  |  |
| unc. Babeliales | 0.06 | 0.01 | |  |  |  |  |  |
| Elusimicrobiota | 0.90 | 0.08 | | 0.13 | 0.01 | 0.03 | 0.06 |  |
| unc. Lineage IV | 0.31 | 0.08 | | 0.12 | 0.01 | 0.02 | 0.06 |  |
| unc. MVP-88 | 0.02 |  | |  |  |  |  |  |
| unc. Elusimicrobia |  |  | |  | 0.01 | 0.01 |  |  |
| unc. Elusimicrobia | 0.01 |  | |  |  |  |  |  |
| unc. Lineage IIa | 0.04 |  | |  |  |  |  |  |
| unc. Lineage IIb | 0.06 |  | | 0.01 |  |  |  |  |
| unc. Lineage IIc | 0.46 |  | |  |  |  |  |  |
| Abditibacteriota |  |  | |  | 0.01 | 0.01 |  |  |
| unc. FBP |  |  | |  | 0.01 | 0.01 |  |  |
| **Bacillota** | **1.84** | 0.48 | | 0.02 | 0.03 | **12.80** | 0.07 | 0.12 |
| unc. Bacillaceae |  |  | |  |  | 0.02 |  |  |
| Ureibacillus | 0.01 |  | |  |  |  |  |  |
| unc. Bacillales | 0.01 |  | |  |  | 0.01 |  |  |
| **Taxon**  Table S1. (continued) | **ODW** | **OIC** | | **OAR** | **OCA** | **OCW** | **OSE1** | **OSE2** |
| Exiguobacterium | 0.08 | 0.03 | |  |  | 0.08 |  |  |
| Paenibacillus | 0.05 |  | |  |  | 0.16 |  | 0.02 |
| unc. Paenibacillaceae | 0.03 |  | |  | 0.01 |  | 0.07 | 0.04 |
| Chryseomicrobium | 0.18 |  | |  |  |  |  |  |
| Jeotgalibacillus |  |  | |  |  | 0.04 |  |  |
| **Paenisporosarcina** | 0.07 | 0.01 | | 0.01 | 0.02 | **3.46** |  | 0.06 |
| unc. Planococcaceae | 0.05 |  | |  |  | 0.22 |  |  |
| Planococcus |  |  | |  |  | 0.17 |  |  |
| Planomicrobium | 0.12 |  | |  |  | 0.13 |  |  |
| Solibacillus | 0.07 |  | |  |  |  |  |  |
| Sporosarcina |  |  | | 0.01 |  |  |  |  |
| Jeotgalicoccus | 0.06 | 0.01 | |  |  | 0.01 |  |  |
| Macrococcus | 0.36 | 0.19 | |  |  | 0.16 |  |  |
| unc. S31 |  |  | |  |  | 0.01 |  |  |
| unc. Staphylococcaceae |  | 0.01 | |  |  |  |  |  |
| Staphylococcus | 0.04 | 0.01 | |  |  | 0.02 |  |  |
| **Aerococcus** | 0.49 | 0.19 | |  |  | **8.23** |  |  |
| Facklamia | 0.06 |  | |  |  |  |  |  |
| Carnobacterium | 0.07 | 0.02 | |  |  | 0.01 |  |  |
| Desemzia | 0.06 | 0.01 | |  |  | 0.01 |  |  |
| unc. Enterococcaceae | 0.01 |  | |  |  |  |  |  |
| unc. Lactobacillales |  |  | |  |  | 0.02 |  |  |
| Anaerococcus |  |  | |  |  | 0.03 |  |  |
| Finegoldia |  |  | |  |  | 0.01 |  |  |
| unc. Lachnospiraceae | 0.01 |  | |  |  |  |  |  |
| Sporacetigenium |  |  | |  |  | 0.01 |  |  |
| Fusobacteriota | 0.03 |  | |  |  |  |  |  |
| Fusobacterium | 0.03 |  | |  |  |  |  |  |
| **Gemmatimonadota** | 0.69 | 0.41 | | **2.63** | 0.39 | **2.13** | **30.22** | **31.03** |
| unc. AKAU4049 |  |  | |  |  |  | 0.83 |  |
| unc. BD2-11 terrestrial group |  |  | |  | 0.01 |  |  |  |
| unc. Gemmatimonadaceae |  | 0.01 | |  | 0.01 | 0.01 | 0.01 |  |
| **unc. Gemmatimonadaceae** | 0.15 | 0.07 | | 0.28 | 0.10 | 0.66 | **6.29** | **12.71** |
| Gemmatimonas | 0.07 | 0.22 | | 0.75 | 0.02 | 0.05 | 0.02 | 0.03 |
| **Gemmatirosa** | 0.07 |  | | 0.25 | 0.03 | 0.05 | 0.48 | **1.40** |
| **unc. Gemmatimonadaceae** | 0.35 | 0.09 | | **1.20** | 0.24 | **1.30** | **21.06** | 0.55 |
| unc. Gemmatimonadetes |  |  | |  |  |  | 0.17 | 0.04 |
| **unc. Longimicrobiaceae** |  |  | | 0.06 |  |  | 0.04 | **15.85** |
| unc. Longimicrobiaceae |  |  | |  |  | 0.01 |  |  |
| **unc. S0134 terrestrial group** | 0.05 | 0.01 | | 0.08 |  | 0.04 | **1.33** | 0.46 |
| Candidatus Hydrogenedentes | 0.02 |  | |  |  |  |  |  |
| unc. Hydrogenedensaceae | 0.02 |  | |  |  |  |  |  |
| Candidatus Latescibacterota |  |  | |  | 0.01 |  | 0.01 |  |
| unc. Latescibacteria |  |  | |  | 0.01 |  | 0.01 |  |
| Candidatus Lindowiibacteriota | 0.04 |  | |  |  |  |  |  |
| unc. Lindowbacteria | 0.04 |  | |  |  |  |  |  |
| Candidatus Margulisiibacteriota | 0.04 |  | |  |  |  |  |  |
| unc. Margulisbacteria | 0.04 |  | |  |  |  |  |  |
| **Nitrospirota** | 0.13 |  | | **1.62** | 0.29 | **2.01** | 0.10 |  |
| **Nitrospira** | 0.13 |  | | **1.62** | 0.29 | **2.01** | 0.10 |  |
| **Candidatus Omnitrophota** | **1.67** |  | |  |  |  |  |  |
| Candidatus Omnitrophus | 0.36 |  | |  |  |  |  |  |
| **unc. Omnitrophicaeota** | **1.28** |  | |  |  |  |  |  |
| unc. Omnitrophicaeota | 0.02 |  | |  |  |  |  |  |
| **Candidatus Patescibacteria** | **14.26** | 0.61 | | **3.87** | **5.98** | **1.63** | **6.28** | 0.43 |
| unc. ABY1 | 0.23 | 0.02 | |  |  |  |  |  |
| Candidatus Buchananbacteria | 0.04 |  | |  |  |  |  |  |
| Candidatus Komeilibacteria | 0.03 |  | |  |  |  |  |  |
| Candidatus Magasanikbacteria | 0.21 |  | |  |  |  |  |  |
| Candidatus Uhrbacteria | 0.04 |  | |  |  |  |  |  |
| **Taxon**  Table S1. (continued) | **ODW** | **OIC** | | **OAR** | **OCA** | **OCW** | **OSE1** | **OSE2** |
| unc. Berkelbacteria | 0.01 |  | |  |  |  | 0.05 |  |
| unc. Absconditabacteriales (SR1) | 0.01 |  | |  |  |  |  |  |
| Candidatus Peregrinibacteria | 0.12 |  | |  |  |  |  |  |
| Candidatus Peribacteria | 0.93 | 0.03 | |  | 0.09 | 0.04 | 0.05 |  |
| unc. Gracilibacteria | 0.10 | 0.12 | |  |  | 0.04 |  |  |
| unc. Gracilibacteria | 0.03 |  | |  |  |  |  |  |
| Candidatus Chisholmbacteria | 0.01 |  | |  |  |  |  |  |
| Candidatus Curtissbacteria | 0.14 |  | |  |  |  |  |  |
| Candidatus Daviesbacteria | 0.03 |  | |  |  |  |  |  |
| Candidatus Levybacteria | 0.08 |  | | 0.21 | 0.06 | 0.05 | 0.24 |  |
| Candidatus Pacebacteria |  |  | |  |  |  | 0.01 |  |
| Candidatus Roizmanbacteria | 0.04 |  | |  |  |  | 0.01 |  |
| Candidatus Woesebacteria | 0.06 |  | |  |  |  | 0.01 |  |
| unc. Microgenomatia |  |  | |  | 0.01 |  |  |  |
| unc. Microgenomatia |  | 0.01 | |  |  |  | 0.02 |  |
| Candidatus Adlerbacteria | 0.41 |  | |  |  |  |  |  |
| **Candidatus Azambacteria** | **1.04** |  | |  | 0.01 |  |  |  |
| Candidatus Campbellbacteria | 0.04 |  | |  |  |  |  |  |
| Candidatus Doudnabacteria | 0.03 | 0.01 | |  |  |  |  |  |
| Candidatus Giovannonibacteria | 0.11 |  | |  |  |  |  |  |
| Candidatus Jorgensenbacteria | 0.09 |  | |  |  |  |  |  |
| Candidatus Kaiserbacteria | 0.57 |  | |  | 0.08 |  | 0.74 | 0.35 |
| Candidatus Liptonbacteria | 0.16 | 0.01 | |  |  |  |  |  |
| Candidatus Lloydbacteria | 0.39 |  | |  |  |  |  |  |
| Candidatus Moranbacteria |  |  | |  |  |  | 0.03 |  |
| **Candidatus Nomurabacteria** | **1.64** | 0.02 | |  | 0.01 |  | 0.05 |  |
| Candidatus Ryanbacteria | 0.17 |  | |  |  |  |  |  |
| Candidatus Staskawiczbacteria | 0.96 |  | |  |  |  |  |  |
| Candidatus Terrybacteria | 0.08 |  | |  |  |  |  |  |
| Candidatus Vogelbacteria | 0.30 |  | |  |  |  |  |  |
| Candidatus Wolfebacteria | 0.04 |  | |  |  |  |  |  |
| **Candidatus Yanofskybacteria** | **1.21** |  | |  |  |  |  |  |
| Candidatus Zambryskibacteria | 0.13 |  | |  |  |  | 0.09 |  |
| unc. GWA2-38-13b | 0.12 | 0.03 | |  |  |  |  |  |
| unc. Parcubacteria | 0.68 |  | |  |  |  | 0.05 |  |
| **unc. Parcubacteria** | **3.21** | 0.09 | | 0.03 | 0.01 | 0.07 | 0.13 | 0.01 |
| unc. Patescibacteria | 0.12 |  | |  |  |  | 0.05 |  |
| unc. Saccharimonadaceae | 0.02 |  | |  |  |  | 0.03 |  |
| **unc. Saccharimonadales** | 0.61 | 0.22 | | **3.42** | **5.62** | **1.38** | **4.69** | 0.06 |
| unc. Saccharimonadales |  | 0.04 | |  | 0.02 | 0.05 |  |  |
| unc. WWE3 |  |  | |  | 0.01 |  |  |  |
| unc. Parcubacteria |  |  | | 0.21 | 0.09 | 0.01 | 0.03 |  |
| **Planctomycetota** | 0.94 | 0.11 | | **2.50** | **4.90** | **1.30** | **2.60** | **3.20** |
| unc. OM190 | 0.01 |  | |  |  |  |  |  |
| unc. CCM11a | 0.03 |  | |  |  | 0.01 |  |  |
| unc. Phycisphaerae |  |  | |  |  |  |  | 0.05 |
| unc. AKYG587 |  |  | |  |  | 0.02 |  |  |
| Algisphaera |  |  | |  |  |  |  | 0.04 |
| unc. I-8 |  |  | |  |  | 0.01 |  |  |
| unc. Phycisphaeraceae |  |  | |  |  |  | 0.02 |  |
| unc. SM1A02 | 0.02 | 0.02 | | 0.11 | 0.26 |  | 0.20 |  |
| unc. Phycisphaeraceae | 0.01 |  | |  |  |  | 0.06 |  |
| unc. Pla1 lineage | 0.01 |  | |  |  |  |  |  |
| Tepidisphaera |  |  | |  | 0.01 |  |  |  |
| unc. Tepidisphaerales |  |  | |  |  |  |  | 0.11 |
| unc. WD2101 soil group | 0.22 | 0.03 | | 0.39 | 0.02 | 0.17 | 0.95 | 0.86 |
| unc. Pla4 lineage | 0.01 |  | |  | 0.01 | 0.03 |  |  |
| Fimbriiglobus | 0.09 | 0.01 | | 0.44 | 0.21 | 0.33 | 0.01 |  |
| Gemmata | 0.07 |  | | 0.24 | 0.12 | 0.11 | 0.26 |  |
| unc. Gemmataceae | 0.01 |  | | 0.01 | 0.01 | 0.03 | 0.03 |  |
| **Taxon**  Table S1. (continued) | **ODW** | **OIC** | | **OAR** | **OCA** | **OCW** | **OSE1** | **OSE2** |
| Zavarzinella | 0.03 |  | |  |  |  |  |  |
| unc. Gemmataceae | 0.08 |  | | 0.87 | 0.05 | 0.04 | 0.39 | 0.02 |
| unc. Isosphaeraceae |  |  | |  |  | 0.01 |  |  |
| unc. Isosphaeraceae | 0.01 | 0.02 | | 0.34 | 0.57 | 0.32 |  | 0.46 |
| Blastopirellula |  |  | |  |  |  | 0.10 |  |
| **unc. Pir4 lineage** | 0.07 | 0.02 | | 0.06 | 0.20 | 0.07 | 0.10 | **1.67** |
| Pirellula | 0.03 |  | | 0.01 |  | 0.01 | 0.16 |  |
| unc. Pirellulaceae | 0.02 |  | |  |  |  | 0.05 |  |
| Rhodopirellula |  |  | |  | 0.01 |  |  |  |
| unc. Pirellulaceae |  |  | |  |  | 0.03 | 0.15 |  |
| unc. Planctomycetacia | 0.01 |  | |  |  |  |  |  |
| Planctomicrobium |  |  | |  |  |  | 0.01 |  |
| unc. SH-PL14 |  |  | |  |  |  | 0.01 |  |
| Planctopirus |  |  | |  |  | 0.01 |  |  |
| Schlesneria | 0.01 |  | |  |  | 0.01 |  |  |
| unc. Planctomycetales | 0.02 |  | |  |  |  |  |  |
| unc. Planctomycetales |  |  | | 0.02 | 0.01 |  | 0.08 |  |
| unc. Planctomycetes | 0.01 |  | |  |  |  |  |  |
| **unc. vadinHA49** | 0.14 | 0.01 | |  | **3.43** | 0.11 |  |  |
| **Pseudomonadota** | **58.08** | **54.43** | | **23.40** | **33.06** | **38.42** | **21.99** | **23.41** |
| unc. Acetobacteraceae |  |  | | 0.01 | 0.01 | 0.04 | 0.01 |  |
| Roseococcus |  |  | |  |  |  |  |  |
| Roseomonas |  |  | |  |  | 0.07 |  |  |
| unc. Alphaproteobacteria | 0.11 | 0.05 | | 0.02 | 0.21 | 0.07 | 0.04 |  |
| **Brevundimonas** | **13.99** | **1.80** | | **1.98** | **7.45** | **1.60** | 0.01 |  |
| Caulobacter |  | 0.02 | |  |  |  |  |  |
| unc. Caulobacteraceae | 0.06 |  | |  | 0.02 |  |  |  |
| unc. Caulobacteraceae |  |  | | 0.17 | 0.13 | 0.03 |  |  |
| unc. PMMR1 |  |  | | 0.06 |  |  |  |  |
| Phenylobacterium |  | 0.01 | |  | 0.01 | 0.16 | 0.01 |  |
| unc. Caulobacterales |  |  | |  |  |  |  |  |
| unc. Hyphomonadaceae |  |  | | 0.01 |  |  |  |  |
| unc. Elsterales |  |  | |  |  |  | 0.01 |  |
| unc. Holosporaceae |  |  | |  |  | 0.17 |  |  |
| unc. Micropepsaceae | 0.02 |  | |  |  |  |  |  |
| unc. NRL2 |  |  | |  |  |  |  |  |
| Candidatus Captivus |  |  | |  | 0.01 | 0.01 | 0.02 |  |
| Candidatus Paracaedibacter |  |  | |  |  | 0.04 |  |  |
| Reyranella | 0.01 |  | | 0.24 | 0.05 | 0.06 | 0.15 |  |
| unc. A0839 |  |  | |  |  | 0.01 |  |  |
| unc. Beijerinckiaceae | 0.02 |  | | 0.03 |  | 0.01 |  |  |
| Bosea |  |  | |  | 0.03 | 0.01 |  |  |
| Methylobacterium | 0.04 |  | |  |  |  |  |  |
| Psychroglaciecola |  |  | | 0.06 | 0.07 | 0.02 |  |  |
| Devosia | 0.03 |  | |  |  | 0.01 |  |  |
| unc. Devosiaceae | 0.03 |  | |  |  |  | 0.02 |  |
| unc. Devosiaceae |  |  | | 0.01 | 0.02 |  | 0.02 |  |
| Pedomicrobium | 0.01 |  | |  |  |  |  |  |
| unc. Methyloligellaceae |  |  | |  |  |  | 0.12 |  |
| unc. Methyloligellaceae |  |  | |  |  |  | 0.04 |  |
| Aliihoeflea | 0.02 |  | | 0.36 | 0.13 | 0.11 |  | 0.04 |
| Rhizobium | 0.01 |  | |  |  | 0.01 |  |  |
| Pseudaminobacter |  |  | |  | 0.03 | 0.01 |  |  |
| unc. Rhizobiaceae | 0.29 | 0.02 | | 0.14 | 0.30 | 0.09 | 0.11 | 0.37 |
| unc. Rhizobiales_Incertae_Sedis | 0.24 |  | | 0.93 | 0.15 | 0.25 | 0.35 |  |
| unc. Rhizobiales | 0.07 | 0.02 | |  | 0.02 | 0.04 | 0.04 | 0.04 |
| unc. Pseudorhodoplanes |  | 0.01 | |  |  |  | 0.01 |  |
| Rhodoplanes | 0.01 |  | |  |  |  |  |  |
| unc. Xanthobacteraceae | 0.04 |  | | 0.09 | 0.01 | 0.03 | 0.01 |  |
| unc. Rhizobiales |  |  | | 0.50 | 0.10 | 0.01 | 0.02 |  |
| **Taxon**  Table S1. (continued) | **ODW** | **OIC** | | **OAR** | **OCA** | **OCW** | **OSE1** | **OSE2** |
| Cereibacter |  | 0.01 | |  |  |  |  |  |
| Paracoccus | 0.02 |  | |  | 0.01 |  |  |  |
| unc. Rhodobacteraceae | 0.01 |  | | 0.05 |  | 0.18 | 0.01 | 0.05 |
| Rubellimicrobium |  |  | |  |  | 0.02 |  |  |
| unc. Rhodospirillaceae |  |  | |  |  |  | 0.02 |  |
| unc. Rhodospirillales | 0.01 |  | |  |  |  | 0.04 |  |
| Candidatus Jidaibacter |  |  | |  | 0.03 |  |  |  |
| Candidatus Megaira |  |  | |  |  | 0.01 |  |  |
| unc. Rickettsiales | 0.04 |  | |  |  |  |  |  |
| unc. SM2D12 | 0.04 |  | |  |  |  |  |  |
| unc. Ellin6055 |  |  | |  |  |  | 0.01 |  |
| Hephaestia |  |  | |  | 0.04 |  |  |  |
| Novosphingobium | 0.03 | 0.02 | |  | 0.05 | 0.01 | 0.01 |  |
| unc. Plot4-2H12 |  |  | |  |  |  | 0.01 |  |
| Qipengyuania | 0.06 |  | |  | 0.01 | 0.04 | 0.04 |  |
| Rhizorhapis | 0.01 |  | | 0.04 | 0.02 | 0.03 | 0.04 |  |
| Sphingoaurantiacus |  |  | |  |  | 0.01 |  |  |
| Sphingobium | 0.01 |  | |  |  |  |  |  |
| **unc. Sphingomonadaceae** | **1.35** | **3.15** | | **6.29** | **10.57** | **5.49** | 0.24 | 0.15 |
| **Sphingomonas** | **3.61** | 0.54 | | 0.45 | **1.50** | **6.59** | **2.33** | 0.01 |
| Sphingopyxis |  |  | |  |  |  |  |  |
| Candidatus Alysiosphaera | 0.01 |  | |  |  |  |  |  |
| unc. Alphaproteobacteria |  |  | |  |  |  | 0.08 | 0.10 |
| unc. Bacteriovoracaceae | 0.01 |  | |  |  |  |  |  |
| Bacteriovorax |  | 0.07 | |  |  |  |  |  |
| Peredibacter | 0.14 | 0.21 | |  | 0.07 | 0.15 | 0.04 | 0.02 |
| unc. Bacteriovoracaceae |  |  | |  | 0.01 |  |  |  |
| **Bdellovibrio** | 0.29 | 0.08 | | 0.01 | **1.46** | 0.19 | 0.10 |  |
| unc. OM27 clade |  |  | | 0.02 |  | 0.05 | 0.04 |  |
| unc. Bradymonadaceae |  |  | |  |  |  |  | 0.14 |
| unc. Deltaproteobacteria | 0.20 | 0.01 | | 0.01 | 0.05 | 0.02 | 0.05 |  |
| unc. Desulfobulbaceae |  |  | |  |  | 0.01 |  |  |
| unc. 27F-1492R | 0.03 |  | | 0.02 |  |  | 0.06 |  |
| Anaeromyxobacter |  |  | |  |  | 0.36 | 0.02 |  |
| Archangium |  |  | |  |  |  |  | 0.01 |
| unc. BIrii41 |  |  | |  |  |  |  |  |
| unc. Blfdi19 | 0.02 |  | | 0.03 | 0.11 | 0.03 | 0.02 |  |
| Haliangium | 0.14 | 0.05 | | 0.12 | 0.07 | 0.31 | 0.20 | 0.01 |
| unc. KD3-10 | 0.02 |  | |  |  |  |  |  |
| unc. Myxococcales | 0.01 |  | |  |  |  |  |  |
| unc. Myxococcales | 0.02 |  | | 0.01 | 0.03 | 0.24 | 0.08 |  |
| Nannocystis | 0.02 |  | |  |  |  | 0.02 |  |
| unc. P3OB-42 | 0.02 |  | | 0.02 | 0.01 |  |  |  |
| Phaselicystis |  |  | |  | 0.01 |  |  |  |
| Pajaroellobacter | 0.01 | 0.02 | | 0.15 | 0.14 | 0.23 | 0.08 |  |
| unc. Polyangiaceae |  |  | |  | 0.01 |  |  |  |
| unc. Sandaracinaceae | 0.02 |  | |  |  |  |  |  |
| Sandaracinus | 0.02 |  | | 0.01 | 0.01 | 0.03 | 0.01 |  |
| unc. bacteriap25 | 0.03 | 0.02 | |  |  | 0.01 |  |  |
| unc. mle1-27 | 0.01 | 0.01 | | 0.01 |  | 0.05 |  |  |
| unc. Myxococcales | 0.01 |  | | 0.05 | 0.26 | 0.15 | 0.03 |  |
| unc. 0319-6G20 | 0.52 | 0.01 | | 0.11 | 0.20 | 0.18 | 0.05 |  |
| unc. 053A03-B-DI-P58 |  | 0.01 | |  |  |  |  |  |
| Oligoflexus |  | 0.01 | |  |  |  |  |  |
| Silvanigrella |  | 0.02 | |  |  |  |  |  |
| unc. Oligoflexaceae | 0.13 | 0.01 | | 0.02 |  | 0.01 | 0.02 |  |
| unc. Oligoflexales | 0.01 |  | |  |  |  |  |  |
| unc. RCP2-54 | 0.02 |  | |  |  |  |  |  |
| unc. SAR324 clade(Marine group B) |  |  | |  |  |  |  | 0.01 |
| Sulfurifustis | 0.03 |  | |  |  |  |  |  |
| **Taxon**  Table S1. (continued) | **ODW** | **OIC** | | **OAR** | **OCA** | **OCW** | **OSE1** | **OSE2** |
| un. Acidiferrobacteraceae |  |  | |  | 0.01 |  |  |  |
| Marinobacter |  |  | |  | 0.01 |  | 0.01 |  |
| unc. Betaproteobacteriales | 0.10 | 0.07 | | 0.02 | 0.03 | 0.05 | 0.05 |  |
| Actimicrobium | 0.08 | 0.02 | |  | 0.01 |  |  |  |
| Aquabacterium |  | 0.03 | |  |  | 0.01 |  |  |
| **unc. Burkholderiaceae** | 0.09 | **1.92** | |  |  |  |  |  |
| **unc. Burkholderiaceae** | **15.67** | **3.16** | | **1.33** | **1.98** | **2.50** | 0.69 |  |
| **Caenimonas** | 0.02 | 0.06 | | 0.10 |  | **1.41** |  |  |
| Comamonas | 0.01 |  | |  |  |  |  | 0.02 |
| Duganella | 0.01 |  | |  |  |  |  |  |
| Herminiimonas |  | 0.01 | |  |  |  |  |  |
| Hydrogenophaga | 0.02 | 0.10 | |  | 0.01 | 0.02 |  |  |
| Lautropia |  |  | | 0.02 | 0.03 | 0.02 | 0.03 | 0.03 |
| Leptothrix |  | 0.03 | |  | 0.08 | 0.02 | 0.28 |  |
| Massilia | 0.08 | 0.96 | |  |  | 0.01 |  |  |
| Methylibium |  |  | |  |  |  | 0.05 | 0.11 |
| Noviherbaspirillum | 0.06 |  | |  |  |  |  |  |
| Paucibacter | 0.02 | 0.01 | |  |  |  |  |  |
| Pelomonas | 0.02 |  | |  |  |  |  | 0.01 |
| **Polaromonas** | 0.07 | **10.72** | |  | 0.21 | 0.05 |  |  |
| Ralstonia | 0.01 |  | |  |  |  |  |  |
| Rhizobacter | 0.13 | 0.01 | |  |  |  |  |  |
| Rhodoferax | 0.03 | 0.03 | |  |  |  |  |  |
| Rugamonas | 0.01 | 0.01 | |  |  |  |  |  |
| Undibacterium | 0.10 |  | |  |  |  |  |  |
| Variovorax | 0.03 | 0.01 | |  |  | 0.74 |  |  |
| unc. s3t2d-1089 |  |  | |  |  |  |  |  |
| **unc. Burkholderiaceae** | 0.20 | 0.01 | | **3.07** | **2.99** | **3.80** | **4.91** | 0.07 |
| Iodobacter | 0.20 |  | |  |  |  |  |  |
| Candidatus Nitrotoga |  |  | |  |  | 0.01 |  |  |
| Thiobacillus |  |  | | 0.02 |  |  |  |  |
| unc. Leeiaceae |  |  | |  | 0.01 |  |  |  |
| unc. Neisseriaceae | 0.02 |  | |  |  |  |  |  |
| unc. Ellin6067 | 0.02 | 0.01 | |  |  |  | 0.21 |  |
| unc. GOUTA6 |  |  | |  | 0.01 |  |  |  |
| unc. IS-44 | 0.04 |  | |  |  |  | 0.57 |  |
| unc. MND1 | 0.04 |  | |  |  | 0.01 | 0.86 |  |
| Nitrosospira |  | 0.05 | | 0.05 | 0.01 | 0.03 | 0.01 | 0.04 |
| unc. mle1-7 | 0.01 |  | |  |  |  |  |  |
| Procabacter |  |  | |  |  |  | 0.04 |  |
| **unc. SC-I-84** | 0.01 | 0.02 | | **2.12** | 0.14 | 0.12 | **4.85** |  |
| unc. TRA3-20 | 0.18 |  | | 0.26 | 0.03 |  | 0.70 | 0.03 |
| **Cellvibrio** | 0.02 | 0.08 | |  | 0.02 | **1.36** |  |  |
| Coxiella | 0.01 |  | |  | 0.01 |  |  |  |
| Aquicella | 0.04 |  | |  |  |  |  |  |
| unc. Diplorickettsiaceae |  | 0.02 | |  |  |  |  |  |
| unc. Diplorickettsiaceae | 0.06 | 0.03 | |  | 0.04 | 0.08 | 0.03 |  |
| unc. EPR3968-O8a-Bc78 | 0.01 |  | |  |  |  |  |  |
| unc. Ga0077536 | 0.01 |  | | 0.52 | 0.01 | 0.01 | 0.02 |  |
| Acidibacter | 0.01 |  | |  |  |  |  |  |
| Candidatus Ovatusbacter | 0.04 |  | |  |  | 0.01 |  |  |
| Unknown Gammaproteobacteria Family | 0.02 | 0.02 | |  |  |  |  |  |
| Unknown Gammaproteobacteria Family | 0.01 |  | |  |  |  |  |  |
| **unc. Gammaproteobacteria** | 0.17 | 0.36 | | **2.68** | **2.18** | 0.78 | 0.07 | **12.36** |
| Legionella | 0.02 | 0.01 | |  | 0.33 | 0.07 |  |  |
| unc. Methylomonaceae | 0.03 |  | | 0.03 |  |  | 0.39 |  |
| unc. Nitrosococcaceae |  |  | |  |  |  | 0.01 |  |
| unc. wb1-P19 | 0.14 |  | |  | 0.06 | 0.03 | 1.52 |  |
| **Taxon** | **ODW** | **OIC** | | **OAR** | **OCA** | **OCW** | **OSE1** | **OSE2** |
| Alcanivorax | 0.01 |  | |  |  |  |  | 0.01 |
| Pseudohongiella |  |  | |  |  |  |  | 0.01 |
| Oceanobacter | 0.01 |  | |  |  |  |  |  |
| unc. PLTA13 |  |  | |  |  |  | 0.01 |  |
| Acinetobacter | 0.64 | 0.02 | |  |  | 0.67 |  |  |
| **Alkanindiges** | **6.00** | **19.71** | |  |  | 0.04 |  |  |
| Cavicella |  | 0.16 | |  |  | 0.11 |  |  |
| Enhydrobacter | 0.01 |  | |  |  |  |  |  |
| unc. Moraxellaceae |  | 0.10 | |  |  | 0.01 |  |  |
| Paraperlucidibaca |  | 0.01 | |  |  |  |  |  |
| Perlucidibaca | 0.01 | 0.20 | |  |  |  |  |  |
| Psychrobacter | 0.01 |  | |  |  |  |  |  |
| Azorhizophilus | 0.01 |  | |  |  |  |  |  |
| unc. Pseudomonadaceae | 0.02 | 0.02 | |  |  |  |  |  |
| **Pseudomonas** | **8.52** | 0.23 | | 0.52 | 0.15 | 0.76 |  |  |
| unc. Pseudomonadales |  | 0.01 | |  |  |  |  |  |
| Alkanibacter |  | 0.01 | |  |  |  |  |  |
| Panacagrimonas | 0.03 |  | |  |  |  | 0.03 |  |
| Polycyclovorans |  |  | |  | 0.04 | 0.06 | 0.04 |  |
| unc. Solimonadaceae | 0.02 |  | |  |  |  |  |  |
| unc. Solimonadaceae | 0.03 | 0.05 | |  |  |  |  |  |
| unc. Steroidobacteraceae | 0.03 |  | |  |  |  |  |  |
| Mizugakiibacter | 0.02 |  | |  |  |  |  |  |
| Pseudofulvimonas |  | 0.02 | | 0.07 | 0.17 | 0.06 | 0.55 | 0.01 |
| unc. Rhodanobacteraceae |  |  | | 0.01 |  |  | 0.37 |  |
| unc. Rhodanobacteraceae | 0.02 |  | |  |  |  | 0.02 |  |
| Arenimonas | 0.20 | 0.53 | |  |  |  |  |  |
| Luteimonas | 0.05 |  | | 0.03 |  | 0.09 | 0.05 | 0.22 |
| **Lysobacter** | 0.46 | **8.52** | | 0.15 | 0.77 | **5.61** | 0.80 | **9.33** |
| Pseudoxanthomonas | 0.07 | 0.26 | | 0.01 | 0.01 | 0.89 |  |  |
| unc. SN8 |  |  | |  |  |  |  |  |
| **Stenotrophomonas** | **1.61** |  | | 0.06 | 0.01 | 0.31 |  |  |
| Thermomonas | 0.04 | 0.07 | |  | 0.01 | 0.01 |  |  |
| unc. Xanthomonadaceae |  | 0.13 | |  | 0.01 | 0.02 |  | 0.06 |
| **unc. Xanthomonadaceae** | 0.04 | 0.37 | | 0.24 | 0.30 | **1.33** | 0.05 | 0.16 |
| unc. Xanthomonadales |  | 0.03 | |  |  | 0.01 | 0.05 |  |
| unc. Proteobacteria | 0.34 | 0.05 | | 0.01 | 0.04 | 0.01 | 0.08 | 0.01 |
| Candidatus Rokuibacteriota | 0.12 | 0.01 | |  |  | 0.05 |  |  |
| unc. MIZ17 | 0.06 |  | |  |  |  |  |  |
| unc. wb1-A12 |  |  | |  |  |  |  |  |
| unc. Rokubacteriales | 0.06 | 0.01 | |  |  | 0.05 |  |  |
| **Verrucomicrobiota** | 0.72 | 0.10 | | **1.43** | 0.35 | 0.76 | 0.26 | 0.05 |
| **Candidatus Udaeobacter** | 0.25 | 0.03 | | **1.32** | 0.16 | 0.16 |  | 0.01 |
| Chthoniobacter | 0.01 | 0.03 | | 0.06 | 0.15 | 0.17 |  |  |
| unc. Chthoniobacteraceae | 0.03 |  | |  | 0.01 |  | 0.03 | 0.03 |
| unc. Methylacidiphilaceae | 0.20 | 0.01 | |  |  |  |  |  |
| unc. Opitutaceae |  |  | |  | 0.01 |  | 0.03 |  |
| Opitutus | 0.05 |  | | 0.03 | 0.01 | 0.01 | 0.05 | 0.01 |
| unc. Pedosphaeraceae | 0.07 |  | | 0.01 | 0.02 | 0.23 | 0.14 | 0.01 |
| unc. Pedosphaeraceae | 0.06 | 0.02 | |  |  | 0.20 |  |  |
| unc. UA11 | 0.04 |  | |  |  |  |  |  |
| Luteolibacter | 0.01 |  | |  |  |  |  |  |
| Verrucomicrobium |  | 0.01 | |  |  |  |  |  |
| Candidatus Eremiobacterota |  |  | | 0.11 | 0.15 | 0.23 |  |  |
| unc. WPS-2 |  |  | | 0.11 | 0.15 | 0.23 |  |  |
| Candidatus Zixiibacteriota | 0.06 |  | |  |  |  |  |  |
| Zixibacteria | 0.06 |  | |  |  |  |  |  |

Table S1. (continued)

Table S2. Relative abundance of identified archaeal sequences at the phylum and the genus level in Obstans Ice Cave (Genera >1% relative abundance in a sample are in bold. Abbreviations: OIC: Obstans ice; OAR: Obstans aragonite; OCA: Obstans calcite; OCW: Obstans cave wall; OSE1: Obstans sediment, unc.: uncultured).

| **Taxon** | **OIC** | **OAR** | **OCA** | **OCW** | **OSE1** |
| --- | --- | --- | --- | --- | --- |
| **Uncultured Archaea** | **4.89** |  | 0.43 |  |  |
| unc. Archaea | **4.89** |  | 0.43 |  |  |
| Diapherotrites | 0.89 |  |  |  |  |
| unc. Iainarchaeales | 0.89 |  |  |  |  |
| **Euryarchaeota** | **24.00** | **99.97** | **98.40** | **98.50** | **8.91** |
| unc. Euryarchaeota |  |  | 0.43 | 0.03 |  |
| Halococcus | 0.89 |  |  |  |  |
| unc. Halobacteriales |  |  |  | 0.12 |  |
| unc. Methanomicrobia ANME-1b |  |  |  |  | **1.98** |
| unc. Methanosarcinales ANME-2a-2b |  |  | 0.12 |  |  |
| unc. Thermoplasmata | **16.00** | **99.97** | **95.33** | **94.10** |  |
| unc. Thermoplasmata | **7.11** |  | **2.52** | **4.25** | **6.93** |
| **Nanoarchaeaeota** | **54.22** | 0.03 |  |  | **91.09** |
| unc. Woesearchaeia | **54.22** | 0.03 |  |  | **91.09** |
| **Nitrososphaerota** | **16.00** |  | **1.17** | **1.50** |  |
| unc. Nitrosopumilaceae | **1.78** |  |  | 0.46 |  |
| Candidatus Nitrocosmicus | **5.78** |  | 0.98 | 0.89 |  |
| unc. Nitrososphaeraceae | **4.44** |  |  |  |  |
| unc. Nitrosotaleaceae | **4.00** |  | 0.18 | 0.15 |  |

Table S3. Sequence numbers, OTU-numbers, coverage, estimated species numbers and diversity indices of the archaeal community in Obstans Ice Cave, based on 1626 subsampled sequences (in parentheses: values corresponding to lower and upper limits of 95% confidence intervals).

| Sample | Number of reads | Number of OTUs | Good's coverage (%) | Chao1 | Shannon | Inverse Simpson's (1/D) |
| --- | --- | --- | --- | --- | --- | --- |
| OIC | 225 | 40 |  |  |  |  |
| OAR | 5958 | 2 | 99,9 | 1.50  (1.50; 0.701) | 0.003  (0; 0.007) | 1.00  (0.99; 1.00) |
| OCA | 1626 | 8 | 100 | 8.00  (8.00; 8.00) | 0.261  (0.213; 0.309) | 1.10  (1.08; 1.12) |
| OCW | 7318 | 13 | 99,8 | 12.5  (11.8; 16.3) | 0.362  (0.309; 0.416) | 1.15  (1.13; 1.18) |
| OSE1 | 101 | 3 |  |  |  |  |

Table S4. Taxonomic characteristics of strains from samples from Obstans Ice Cave (OAR: Obstans aragonite; OCA: Obstans calcite; OCW: Obstans cave wall; OSE1: Obstans sediment).

| **Strain** | **Closest identified relative in EzBioCloud (accession number)** | | | **% similarity** | **Medium** |
| --- | --- | --- | --- | --- | --- |
| **Actinomycetota** | | | | | |
| OCW-305 | *Arthrobacter ginsengisoli* | DCY81(T) | KF212463 | 99.17 | OB4M1.5 |
| OCW-113 | *Arthrobacter glacialis* | HLT2-12-2(T) | JX949500 | 99.31 | CM |
| OCW-2 |  |  |  | 99.31 | 10% R2A |
| OCW-7 |  |  |  | 99.36 | 10% R2A |
| OCW-8 |  |  |  | 99.34 | 10% R2A |
| OCW-301 |  |  |  | 99.34 | OB4M1.5 |
| OCW-303 |  |  |  | 99.28 | OB4M1.5 |
| OCW-304 |  |  |  | 99.28 | OB4M1.5 |
| OCW-306 |  |  |  | 99.28 | OB4M1.5 |
| OCW-313 |  |  |  | 99.31 | OB4M1.5 |
| OCW-314 |  |  |  | 99.29 | OB4M1.5 |
| OAR-321 | *Arthrobacter monumenti* | LMG 19502(T) | AJ315070 | 97.38 | OB4M1.5 |
| OCW-106 | *Arthrobacter psychrochitiniphilus* | GP3(T) | AJ810896 | 99.20 | CM |
| OCW-312 | *Arthrobacter tumbae* | LMG 19501(T) | AJ315069 | 98.35 | OB4M1.5 |
| OSE1-324 |  |  |  | 97.97 | OB4M1.5 |
| OSE1-325 |  |  |  | 97.83 | OB4M1.5 |
| OCA-318 | *Janibacter terrae* | NBRC 107853(T) | BCUV01000003 | 100 | OB4M1.5 |
| OCA-21 | *Micrococcus antarcticus* | T2(T) | AJ005932 | 99.80 | 10% R2A |
| OCA-122 |  |  |  | 99.79 | CM |
| OCA-22 | *Nocardioides allogilvus* | CFH 30205(T) | MG800321 | 99.44 | 10% R2A |
| OCA-116 |  |  |  | 99.41 | CM |
| OCA-121 |  |  |  | 99.27 | CM |
| OCA-124 |  |  |  | 99.60 | CM |
| OCA-138 |  |  |  | 98.22 | CM |
| OCA-15 | *Paeniglutamicibacter sulfureus* | DSM 20167(T) | X83409 | 99.51 | 10% R2A |
| OCA-16 | *Pseudarthrobacter oxydans* | DSM 20119(T) | X83408 | 95.39 | 10% R2A |
| OCA-119 | *Pseudarthrobacter siccitolerans* | 4J27(T) | CAQI01000001 | 99.37 | CM |
| OCW-109 | *Rhodococcus cerastii* | C5(T) | FR714842 | 100 | CM |
| OCW-310 |  |  |  | 99.52 | OB4M1.5 |
| OCW-3 | *Rhodococcus fascians* | LMG 3623(T) | JMEN01000010 | 99.88 | 10% R2A |
| OCW-302 |  |  |  | 99.81 | OB4M1.5 |
| OSE1-128 |  |  |  | 99.14 | CM |
| OCA-317 | *Rhodococcus sovatensis* | H004(T) | KU189221 | 98.74 | OB4M1.5 |
| OAR-137 | *Streptomyces beijiangensis* | NBRC 100044(T) | AB249973 | 98.82 | CM |
| OSE1-26 |  |  |  | 97.39 | 10% R2A |
| OSE1-133 |  |  |  | 98.51 | CM |
| OCA-120 | *Streptomyces drozdowiczii* | NBRC 101007(T) | AB249957 | 98.63 | CM |
| OAR-12 |  |  |  | 98.65 | 10% R2A |
|  |  |  |  |  |  |
|  |  |  |  |  |  |
|  |  |  |  |  |  |
| Table S4. (continued) | | | | | |
| **Strain** | **Closest identified relative in EzBioCloud (accession number)** | | | **% similarity** | **Medium** |
| **Bacillota** | | | | | |
| OAR-215 | *Priestia (Bacillus) aryabhattai* | B8W22(T) | EF114313 | 100 | R2A |
| OAR-221 | *Priestia (Bacillus) megaterium* | NBRC 15308(T) | JJMH01000057 | 100 | R2A |
| OAR-214 |  |  |  | 100 | R2A |
| OAR-216 |  |  |  | 99.40 | R2A |
| OAR-217 |  |  |  | 100 | R2A |
| OAR-219 |  |  |  | 99.53 | R2A |
| OAR-220 |  |  |  | 99.70 | R2A |
| OAR-222 |  |  |  | 99.70 | R2A |
| OAR-223 |  |  |  | 98.27 | R2A |
| OAR-224 |  |  |  | 99.71 | R2A |
| OAR-407 | *Peribacillus simplex* | NBRC 15720(T) | BCVO01000086 | 99.72 | B4 |
| OAR-409 |  |  |  | 100 | B4 |
| OAR-206 |  |  |  | 100 | R2A |
| OAR-207 |  |  |  | 100 | R2A |
| OAR-211D |  |  |  | 96.48 | R2A |
| OAR-213 |  |  |  | 100 | R2A |
| OAR-401 |  |  |  | 100 | B4 |
| OAR-406 |  |  |  | 100 | B4 |
| OAR-201 |  |  |  | 100 | R2A |
| OAR-202 |  |  |  | 100 | R2A |
| OAR-208 |  |  |  | 100 | R2A |
| OAR-210 |  |  |  | 100 | R2A |
| OAR-211B |  |  |  | 100 | R2A |
| OAR-402 |  |  |  | 99.56 | B4 |
| OAR-218 |  |  |  | 99.58 | R2A |
| OAR-203 |  |  |  | 100 | R2A |
| OAR-211C |  |  |  | 99.90 | R2A |
| OAR-408 |  |  |  | 100 | B4 |
| OCA-141 | *Jeotgalibacillus marinus* | DSM 1297(T) | AJ237708 | 99.09 | CM |
| OCA-315 |  |  |  | 99.15 | OB4M1.5 |
| OSE1-130 | *Paenibacillus luteus* | R-3(T) | KY674516 | 98.09 | CM |
| OAR-9 | *Paenisporosarcina antarctica* | N-05(T) | EF154512 | 98.88 | 10% R2A |
| OAR-115 |  |  |  | 98.93 | CM |
| OCW-108 | *Paenisporosarcina indica* | PN2T(T) | FN397659 | 99.49 | CM |
| OSE1-308 |  |  |  | 98.67 | OB4M1.5 |
| OCW-111 | *Planococcus salinarum* | DSM 23820(T) | MBQG01000128 | 97.31 | CM |
| OCA-17 | *Planococcus chinensis* | DX3-12(T) | AJ697862 | 99.69 | 10% R2A |
| OCW-6 | *Planococcus koreensis* | JG07(T) | AF144750 | 100 | 10% R2A |
| OCW-311 |  |  |  | 100 | OB4M1.5 |
| OSE1-134 | *Sporosarcina globispora* | DSM 4(T) | X68415 | 99.90 | CM |
|  |  |  |  |  |  |
|  |  |  |  |  |  |
| Table S4. (continued) | | | | | |
| **Strain** | **Closest identified relative in EzBioCloud (accession number)** | | | **% similarity** | **Medium** |
| OCW-107 | *Paenisporosarcina macmurdoensis* | CMS 21w(T) | AJ514408 | 98.80 | CM |
| OAR-10 |  |  |  | 97.22 | 10% R2A |
| OAR-13 |  |  |  | 97.35 | 10% R2A |
| OAR-114 |  |  |  | 97.35 | CM |
| OCA-23 |  |  |  | 99.40 | 10% R2A |
| OCA-316 |  |  |  | 99.68 | OB4M1.5 |
| OCW-1 |  |  |  | 99.69 | 10% R2A |
| OCW-110 |  |  |  | 98.57 | CM |
| OCW-136 |  |  |  | 98.79 | CM |
| OSE1-131 |  |  |  | 98.76 | CM |
| OSE1-135 |  |  |  | 99.61 | CM |
| OSE1-322 |  |  |  | 99.57 | OB4M1.5 |
| **Bacteroidota** | | | | | |
| OCA-18 | *Flavobacterium psychroterrae* | CCM 8827(T) | MH100901 | 97.88 | 10% R2A |
| OCA-19 |  |  |  | 98.32 | 10% R2A |
| OSE1-132 | *Flavobacterium sinopsychrotolerans* | 0533(T) | FJ654474 | 98.75 | CM |
| OCW-101 | *Hymenobacter tenuis* | POB6(T) | KY196418 | 99.40 | CM |
| **Pseudomonadota** | | | | | |
| OCA-139 | *Brevundimonas basaltis* | J22(T) | EU143355 | 99.13 | CM |
| OCA-142 | *Brevundimonas subvibrioides* | ATCC 15264(T) | ADBM01000034 | 99.00 | CM |
| OCA-117 | *Brevundimonas variabilis* | ATCC 15255(T) | AJ227783 | 98.84 | CM |
| OCA-123 | *Devosia psychrophila* | Cr7-05(T) | LAPV01000071 | 99.38 | CM |
| OCW-102 | *Massilia aurea* | AP13(T) | AM231588 | 99.37 | CM |
| OAR-32 | *Massilia eurypsychrophila* | JCM 30074(T) | PDOC01000073 | 97.85 | 10% R2A |
| OAR-33 |  |  |  | 97.94 | 10% R2A |
| OCA-20 |  |  |  | 97.81 | 10% R2A |
| OCA-31 | *Massilia niabensis* | 5420S-26(T) | EU808006 | 98.28 | 10% R2A |
| OCA-14 | *Massilia psychrophila* | JCM 30813(T) | PDOB01000071 | 97.25 | 10% R2A |
| OCW-104 | *Massilia timonae* | CCUG 45783(T) | AGZI01000009 | 98.04 | CM |
| OCW-103 | *Pseudomonas extremaustralis* | 14-3(T) | AHIP01000073 | 99.08 | CM |
| OCW-105 | *Pseudomonas frederiksbergensis* | JAJ28(T) | AJ249382 | 99.16 | CM |
| OCA-118 |  |  |  | 99.48 | CM |
| OCA-320 |  |  |  | 98.82 | OB4M1.5 |
| OCW-112 | *Pseudomonas mandelii* | NBRC 103147(T) | BDAF01000092 | 99.71 | CM |

Table S5. Metabolic characteristics of selected strains isolated from Obstans Ice Cave. Legend: -: negative; +: weak positive, ++: positive, +++: strong positive, MR-VP: metilred and Voges-Proskauer reaction.

| **Strain** | | **Nitrate reduction to nitrite** | **Nitrate reduction to ammonia** | **Nitrate reduction to nitrogen** | **Urease** | **H_2_S production from peptone** | **NH_3_ production from peptone** | **Peptone hydrolysis** | **Oxidation of D-glucose** | **Fermentation of D-glucose** | **MR-VP** |
| --- | --- | --- | --- | --- | --- | --- | --- | --- | --- | --- | --- |
| *Arthrobacter glacialis* | OCW-113 | ++ | + | - | +++ | + | - | - | - | - | - |
| *Arthrobacter glacialis* | OCW-2 | + | + | - | ++ | + | - | - | - | - | - |
| *Arthrobacter glacialis* | OCW-313 | ++ | - | + | ++ | - | +++ | - | - | - | - |
| *Peribacillus simplex* | OAR-407 | +++ | - | + | - | - | + | - | + | - | - |
| *Peribacillus simplex* | OAR-202 | +++ | - | + | - | - | - | - | + | - | - |
| *Peribacillus simplex* | OAR-206 | +++ | - | - | - | - | - | - | + | - | - |
| *Planomicrobium koreense* | OCW-311 | +++ | - | + | + | - | - | ++ | - | - | - |
| *Hymenobacter tenuis* | OCW-101 | - | +++ | ++ | + | ++ | ++ | - | ++ | + | - |
| *Planococcus salinarum* | OCW-111 | - | ++ | + | + | - | + | + | - | - | - |
| *Arthrobacter ginsengisoli* | OCW-305 | +++ | +++ | + | - | - | + | + | - | - | - |
| *Paeniglutamicibacter sulfureus* | OCA-15 | ++ | + | - | - | + | ++ | + | - | - | - |
| *Massilia eurypsychrophila* | OCA-14 | - | - | - | - | - | ++ | + | - | - | - |

Table S6. Comparison of bacterial and archaeal taxa detected in various ice cave habitat types.

|  | **Limestone bedrock** | | | | | | | **Volcanic bedrock** | | |
| --- | --- | --- | --- | --- | --- | --- | --- | --- | --- | --- |
| Locality | Obstans Ice Cave (Austria, this study) | | | Scărişoara Ice Cave (Romania) ^a,b^ | Paradana Ice Cave (Slovenia) ^c^ | A294 Ice Cave (Central Pyrenees, Iberia) ^d^ | Morgana Cave (Italy) ^e^ | Mauna Loa ice caves (Hawaii) ^f^ | | Mt. Erebus ice caves (Antarctica) ^g^ |
| Habitat type | Ice and drip water | Carbonate precipitates | clastic sediments | Ice | Frozen lake | Ice | Vermicular deposits | Calcite, gypsum and silica precipitates | Ice | Clastic sediments |
| **Pseudomonadota** | **+** | **+** | **+** | **+** |  | **+** | **+** |  | **+** | **+** |
| *Sphingomonas* |  | **+** |  |  | **+** | **+** |  |  |  | **+** |
| unc. Solirubrobacterales |  | **+** |  |  |  |  |  |  |  | **+** |
| unc. Burkholderiales | **+** |  |  |  |  |  | **+** |  |  | **+** |
| *Pseudomonas* | **+** |  |  | **+** | **+** |  |  |  |  |  |
| *Stenotrophomonas* |  |  |  | **+** |  |  |  |  |  |  |
| *Brevundimonas* | **+** |  |  | **+** |  |  |  |  |  |  |
| *Polaromonas* | **+** |  |  | **+** | **+** |  |  |  |  |  |
| *Lysobacter* | **+** | **+** |  |  | **+** | **+** |  |  |  |  |
| unc. Nitrosomonadaceae |  |  |  |  |  |  | **+** |  |  |  |
| unc. wb1-P19 |  |  |  |  |  |  | **+** |  |  |  |
| unc. Gammaproteobacteria |  |  | **+** |  | **+** |  |  |  |  |  |
| **Actinomycetota** |  | **+** | **+** | **+** |  | **+** | **+** | **+** | **+** | **+** |
| *Pseudonocardia* |  | **+** |  | **+** | **+** |  |  |  |  |  |
| *Gaiella* |  |  |  |  | **+** |  | **+** |  |  |  |
| *Arthrobacter* |  |  |  |  |  | **+** |  |  |  |  |
| Unc. Acidimicrobiia |  |  | **+** |  |  |  | **+** | **+** |  |  |
| unc. Rubrobacteria |  |  |  |  |  |  | **+** |  |  |  |
| **Acidobacteriota** |  | **+** | **+** |  |  |  | **+** |  |  | **+** |
| unc. Blastocatellaceae |  |  |  |  |  |  | **+** |  |  |  |
| *Blastocatella* |  | **+** |  |  |  |  |  |  |  | **+** |
| unc. Pyrinomonadaceae RB41 |  | **+** |  |  |  |  | **+** |  |  | **+** |
| **Bacteroidota** | **+** | **+** |  |  | **+** | **+** |  |  | **+** | **+** |
| *Pedobacter* |  |  |  | **+** | **+** | **+** |  | **+** |  |  |
| *Flavobacterium* | **+** |  |  | **+** | **+** |  |  |  |  |  |
| **Gemmatimonadota** |  |  | **+** |  |  |  |  |  |  |  |
| **Cyanobacteria** |  |  |  | **+** | **+** |  |  |  |  | **+** |
| **Chloroflexota** |  |  |  | **+** |  |  |  |  |  | **+** |
| **Euryarchaeota** | **+** | **+** | **+** | **+** |  |  | **+** |  | **+** |  |
| **Nanoarchaeota** | **+** |  | **+** |  |  |  | **+** |  |  |  |
| **Nitrososphaerota** | **+** |  |  |  |  |  | **+** | **+** | **+** |  |

References:

a: Iţcuş, C. *et al.* Bacterial and archaeal community structures in perennial cave ice. *Sci. Rep.* **8**, 15671; 10.1038/s41598-018-34106-2 (2018).

b: Paun, V. I. *et al.* Total and potentially active bacterial communities entrapped in a late glacial through holocene ice core from Scărişoara Ice Cave, Romania. *Front. Microbiol.* **10**, 1193; 10.3389/fmicb.2019.01193 (2019).

c: Mulec, J. *et al.* Microbiota entrapped in recently-formed ice: Paradana Ice Cave, Slovenia. *Sci. Rep.* **11**, 1993; 10.1038/s41598-021-81528-6 (2021).

d: Ruiz-Blas, F. *et al.* The hidden microbial ecosystem in the perennial ice from a Pyrenean ice cave. *Front. Microbiol.* **14**, 1110091; 10.3389/fmicb.2023.1110091 (2023).

e: Jurado, V. *et al.* Microbial communities in vermiculation deposits from an alpine cave. *Front. Earth Sci.* **8**, 586248; 10.3389/feart.2020.586248 (2020).

f: Teehera, K. B. *et al.* Cryogenic minerals in Hawaiian lava tubes: a geochemical and microbiological exploration. *Geomicrobiol. J.* **35**, 227–241 (2018).

g: Tebo, B. M. *et al.* Microbial communities in dark oligotrophic volcanic ice cave ecosystems of Mt. Erebus, Antarctica. *Front. Microbiol.* **6**, 179; 10.3389/fmicb.2015.00179 (2015).

Table S7. Calcium carbonate precipitation capacity of bacterial strains incubated at different temperatures and Mg^2+^ content (n.d.: no data).

| **Closest identified relative** | **Strain** | **B4 10°C** | | **B4 21°C** | | **B4M1 10°C** | | **B4M1 21°C** | |
| --- | --- | --- | --- | --- | --- | --- | --- | --- | --- |
|  |  | **Content (%)** | **Mineral** | **Content (%)** | **Mineral** | **Content (%)** | **Mineral** | **Content (%)** | **Mineral** |
| *Arthrobacter ginsengisoli* | OCW-305 | 0 | n.d. | 0 | n.d. | 0 | n.d. | 12 | n.d. |
| *Arthrobacter glacialis* | OCW-313 | n.d. | n.d. | n.d. | n.d. | 0 | n.d. | 8 | n.d. |
|  | OCW-301 | n.d. | n.d. | n.d. | n.d. | 3 | n.d. | 8 | n.d. |
|  | OCW-113 | n.d. | n.d. | n.d. | n.d. | 0 | n.d. | 4 | n.d. |
|  | OCW-2 | 0 | n.d. | 0 | n.d. | 0 | n.d. | 17 | Mg-calcite, aragonite |
| *Peribacillus simplex* | OAR-202 | 10 | calcite | 22 | calcite | 58 | Mg-calcite | 2 | Mg-calcite |
|  | OAR-206 | 10 | calcite | 13 | calcite | 0 | n.d. | 0 | n.d. |
|  | OAR-211D | 0 | n.d. | 11 | n.d. | 0 | n.d. | 0 | n.d. |
|  | OAR-406 | 0 | n.d. | 0 | n.d. | 0 | n.d. | 0 | n.d. |
|  | OAR-407 | 16 | n.d. | 0 | n.d. | 0 | n.d. | 2 | n.d. |
| *Hymenobacter tenuis* | OCW-101 | n.d. | n.d. | n.d. | n.d. | 3 | Mg-calcite, aragonite | 5 | Mg-calcite, aragonite |
| *Jeotgalibacillus marinus* | OCA-315 | n.d. | n.d. | n.d. | n.d. | 0 | n.d. | 8 | n.d. |
| *Massilia aurea* | OCW-102 | n.d. | n.d. | n.d. | n.d. | 0 | n.d. | 0 | n.d. |
| *Massilia eurypsychrophila* | OCA-14 | n.d. | n.d. | n.d. | n.d. | 0 | n.d. | 71 | n.d. |
| *Micrococcus antarcticus* | OCA-122 | n.d. | n.d. | n.d. | n.d. | 0 | n.d. | 0 | n.d. |
| *Paeniglutamicibacter sulfureus* | OCA-15 | 64 | n.d. | 25 | calcite | 3 | Mg-calcite | 3 | Mg-calcite |
| *Paenisporosarcina macmurdoensis* | OCW-107 | n.d. | n.d. | n.d. | n.d. | 0 | n.d. | 37 | n.d. |
|  | OCW-1 | n.d. | n.d. | 0 | n.d. | 0 | n.d. | 0 | n.d. |
| *Planococcus salinarum* | OCW-111 | n.d. | n.d. | n.d. | n.d. | 0 | n.d. | 2 | n.d. |
| *Planomicrobium koreense* | OCW-311 | 0 | n.d. | n.d. | n.d. | 0 | n.d. | 0 | n.d. |
| *Pseudomonas extremaustralis* | OCW-103 | n.d. | n.d. | n.d. | n.d. | 5 | n.d. | 8 | n.d. |
| *Rhodococcus cerastii* | OCW-310 | 4 | n.d. | n.d. | n.d. | 50 | n.d. | 17 | n.d. |
| *Rhodococcus fascians* | OCW-3 | n.d. | n.d. | n.d. | n.d. | 80 | n.d. | 0 | n.d. |

Table S8. Time-dependent alteration of acidic-basic characteristics of B4M1 medium supplemented with cresol red and inoculated with bacterial strains at 21 °C. Legend: 0: no change/weak acidification; d: diameter of pH change; 1: d < 1 cm (pale pink); 2: d < 1 cm (reddish purple); 3: 1 cm < d < 2 cm (reddish purple); 4: 2 cm < d < 3 cm (reddish purple); 5: d > 3 cm (reddish purple).

| **Closest identified relative** | **Strain** | **1** | **2** | **3** | **4** | **5** | **6** | **7** | **8** | **9** | **10** | **11** | **12** | **13** | **14** | **15** | **16** | **17** | **18** | **19** | **20** | **21** | **22** | **23** | **24** | **25** | **26** | **27** | **28** | **29** | **days** |
| --- | --- | --- | --- | --- | --- | --- | --- | --- | --- | --- | --- | --- | --- | --- | --- | --- | --- | --- | --- | --- | --- | --- | --- | --- | --- | --- | --- | --- | --- | --- | --- |
| *Planomicrobium koreense* | AMS-311 | 0 | 0 | 0 | 0 | 0 | 0 | 0 | 0 | 0 | 0 | 0 | 0 | 0 | 0 | 0 | 0 | 0 | 0 | 0 | 0 | 0 | 0 | 0 | 0 | 0 | 0 | 0 | 0 | 0 |  |
| *Paeniglutamicibacter sulfureus* | AMC-15 | 0 | 0 | 1 | 3 | 3 | 3 | 4 | 4 | 4 | 4 | 4 | 4 | 5 | 5 | 5 | 5 | 5 | 5 | 5 | 5 | 5 | 5 | 5 | 5 | 5 | 5 | 5 | 5 | 5 |  |
| *Peribacillus simplex* | AMA-202 | 3 | 3 | 3 | 3 | 4 | 4 | 4 | 4 | 5 | 5 | 5 | 5 | 5 | 5 | 5 | 5 | 5 | 5 | 5 | 5 | 5 | 5 | 5 | 5 | 5 | 5 | 5 | 5 | 5 |  |
| *Arthrobacter glacialis* | AMS-113 | 0 | 0 | 0 | 0 | 0 | 0 | 0 | 0 | 0 | 0 | 0 | 0 | 0 | 0 | 0 | 0 | 0 | 0 | 0 | 0 | 0 | 0 | 0 | 0 | 0 | 0 | 0 | 0 | 0 |  |
| *Arthrobacter glacialis* | AMS-313 | 0 | 0 | 0 | 0 | 0 | 0 | 0 | 0 | 0 | 0 | 0 | 2 | 2 | 2 | 3 | 3 | 4 | 4 | 4 | 5 | 5 | 5 | 5 | 5 | 5 | 5 | 5 | 5 | 5 |  |
| *Arthrobacter glacialis* | AMS-2 | 0 | 0 | 0 | 0 | 0 | 0 | 0 | 0 | 0 | 0 | 0 | 0 | 0 | 0 | 0 | 0 | 0 | 0 | 0 | 0 | 0 | 0 | 0 | 0 | 0 | 0 | 0 | 0 | 0 |  |

Table S9. Time-dependent alteration of acidic-basic characteristics of B4M1 medium supplemented with cresol red and inoculated with bacterial strains at 10 °C. Legend: 0: no change/weak acidification; d: diameter of pH change; 1: d < 1 cm (pale pink); 2: d < 1 cm (reddish purple); 3: 1 cm < d < 2 cm (reddish purple); 4: 2 cm < d < 3 cm (reddish purple); 5: d > 3 cm (reddish purple).

| **Closest identified relative** | **Strain** | **4** | **5** | **6** | **8** | **9** | **13** | **14** | **15** | **19** | **23** | **26** | **35** | **40** | **44** | **49** | **57** | **61** | **64** | **68** | **83** | **97** | **104** | **107** | **113** | **118** | **125** | **132** | **134** | **days** |
| --- | --- | --- | --- | --- | --- | --- | --- | --- | --- | --- | --- | --- | --- | --- | --- | --- | --- | --- | --- | --- | --- | --- | --- | --- | --- | --- | --- | --- | --- | --- |
| *Planomicrobium koreense* | AMS-311 | 0 | 0 | 0 | 0 | 0 | 0 | 0 | 0 | 0 | 0 | 0 | 0 | 0 | 0 | 0 | 0 | 0 | 0 | 0 | 0 | 0 | 0 | 0 | 0 | 0 | 0 | 0 | 0 |  |
| *Paeniglutamicibacter sulfureus* | AMC-15 | 0 | 1 | 1 | 1 | 3 | 3 | 3 | 3 | 3 | 4 | 5 | 5 | 5 | 5 | 5 | 5 | 5 | 5 | 5 | 5 | 5 | 5 | 5 | 5 | 5 | 5 | 5 | 5 |  |
| *Peribacillus simplex* | AMA-202 | 0 | 0 | 0 | 0 | 0 | 1 | 2 | 3 | 3 | 4 | 5 | 5 | 5 | 5 | 5 | 5 | 5 | 5 | 5 | 5 | 5 | 5 | 5 | 5 | 4 | 4 | 4 | 4 |  |
| *Arthrobacter glacialis* | AMS-113 | 0 | 0 | 0 | 0 | 0 | 0 | 0 | 0 | 0 | 0 | 0 | 0 | 0 | 0 | 0 | 0 | 2 | 0 | 0 | 0 | 0 | 0 | 0 | 0 | 0 | 0 | 0 | 0 |  |
| *Arthrobacter glacialis* | AMS-313 | 0 | 0 | 0 | 0 | 0 | 0 | 0 | 0 | 0 | 0 | 0 | 2 | 3 | 4 | 4 | 5 | 5 | 5 | 5 | 0 | 5 | 5 | 3 | 5 | 5 | 3 | 0 | 5 |  |
| *Arthrobacter glacialis* | AMS-2 | 0 | 0 | 0 | 0 | 0 | 0 | 0 | 0 | 0 | 0 | 0 | 0 | 0 | 0 | 0 | 0 | 0 | 0 | 0 | 0 | 0 | 0 | 0 | 0 | 0 | 0 | 0 | 0 |  |

Table S10. SEM EDS analyses of carbonates precipitated by *Peribacillus simplex* OAR-202 on media with various Mg^2+^/Ca^2+^ molar ratios (n.d.: no data).

| **Measures** | 1 | 2 | 3 |
| --- | --- | --- | --- |
| **B4 medium (Ca^2+^=1)** | | | |
| **Mg (atom%)** | 0.3 | 0 | 0.15 |
| **Ca (atom%)** | 11.39 | 14.78 | 11.46 |
| **Sum** | 11.69 | 14.78 | 11.61 |
| **Mg (mol%)** | 2.6 | 0.0 | 1.3 |
| **Mean Mg (mol%)** | 1.3 | | |
| **B4M0.25 medium (Mg^2+^/Ca^2+^=0.25)** | | | |
| **Mg (atom%)** | 0.59 | 0.64 | 0.55 |
| **Ca (atom%)** | 6.87 | 5.75 | 3.53 |
| **Sum** | 7.46 | 6.39 | 4.08 |
| **Mg (mol%)** | 7.9 | 10.0 | 13.5 |
| **Mean Mg (mol%)** | 10.5 | | |
| **B4M0.5 medium (Mg^2+^/Ca^2+^=0.5)** | | | |
| **Mg (atom%)** | 0.38 | 0.51 | 0.5 |
| **Ca (atom%)** | 6.12 | 6.18 | 6.05 |
| **Sum** | 6.5 | 6.69 | 6.55 |
| **Mg (mol%)** | 5.8 | 7.6 | 7.6 |
| **Mean Mg (mol%)** | 7.0 | | |
| **B4M1 medium (Mg^2+^/Ca^2+^=1)** | | | |
| **Mg (atom%)** | 1.03 | 0.99 | 0.55 |
| **Ca (atom%)** | 8.37 | 8.42 | 8.1 |
| **Sum** | 9.4 | 9.41 | 8.65 |
| **Mg (mol%)** | 11.0 | 10.5 | 6.4 |
| **Mean Mg (mol%)** | 9.3 | | |
| **B4M1.5 medium (Mg^2+^/Ca^2+^=1.5)** | | | |
| **Mg (atom%)** | 0.81 | 0.51 | n.d. |
| **Ca (atom%)** | 4.66 | 8.13 | n.d. |
| **Sum** | 5.47 | 8.64 | n.d. |
| **Mg (mol%)** | 14.8 | 5.9 | n.d. |
| **Mean Mg (mol%)** | 10.4 | | |

Table S11. Composition of media used in carbonate precipitation experiments.

| Medium | B4 | B4M0.25 | B4M0.5 | B4M1 | B4M1.5 |
| --- | --- | --- | --- | --- | --- |
| Mg^2+^/Ca^2+^ molar ratio | - | 0.25 | 0.5 | 1 | 1.5 |
| calcium acetate (g/l) | 2.5 | 2.5 | 2.5 | 2.5 | 2.5 |
| MgSO_4_ × 7H_2_O(g/l) | - | 0.87 | 1.75 | 3.5 | 5.2 |
| glucose (g/l) | 5.0 | 5.0 | 5.0 | 5.0 | 5.0 |
| yeast extract (g/l) | 4.0 | 4.0 | 4.0 | 4.0 | 4.0 |
| agar-agar (g/l) | 20 | 20 | 20 | 20 | 20 |
